# Tumor-Centric Immune Crosstalk in Colorectal and Gastric Cancer: Molecular Mechanisms Linking Tumor Cells to Macrophages and CD8+ T Lymphocytes

**DOI:** 10.3390/ijms27209346

**Published:** 2026-10-21

**Authors:** Andrei Pascu, Victor Ianole, Mihai Danciu

**Affiliations:** 1 Department of Morpho-Functional Sciences I, “Grigore T. Popa” University of Medicine and Pharmacy Iași, 16 University Street, 700115 Iasi, Romania; pascu.andrei@email.umfiasi.ro (A.P.); mihada2@yahoo.com (M.D.); 2 Pathology Department, “Sf. Spiridon” University Emergency County Hospital, 1 Independentei Avenue, 700111 Iasi, Romania; 3 Pathology Department, “Prof. Dr. Nicolae Oblu” Emergency Clinical Hospital, 2 Ateneului Street, 700309 Iasi, Romania

**Keywords:** colorectal cancer, gastric cancer, tumor microenvironment, tumor–immune crosstalk, CD8+ T-cells, tumor-associated macrophages, immune evasion

## Abstract

Digestive malignancies remain a major cause of cancer-related mortality, with tumor progression (TP) being strongly influenced by the complex interactions between tumor cells (TCs) and their microenvironment. This review summarizes recently described mechanisms of interactions between tumor cells, macrophages and CD8+ T lymphocytes in colorectal and gastric cancer. PubMed and Web of Science were searched for studies published between 2020 and 2026, with the final search being conducted on 8 July 2026. Experimental and clinical studies, together with relevant reviews, were considered to examine these interactions and their therapeutic implications. The evidence shows a bilateral crosstalk in which tumor cells influence immune cell phenotype and function, while immune cells can further support or restrict tumor progression. These interactions involve receptor-ligand systems and paracrine signaling, followed by changes in intracellular pathways regulating proliferation, invasion, macrophage polarization and CD8+ T-cell exhaustion. Considerable methodological heterogeneity among studies limits a direct comparison of the reported mechanisms. Several therapeutic approaches targeting these interactions are currently being investigated in early-phase clinical trials. However, many components of tumor–immune crosstalk and their connections remain incompletely understood. A further characterization of these mechanisms may help identify therapeutic strategies able to interfere with the immunosuppressive microenvironment of colorectal and gastric cancers.

## 1. Introduction

### 1.1. Current Global Incidence and Future Projections

Cancer is a disease with a severe medical, social, and psychological impact, with the lifetime probability of developing a form of cancer ranging from 39% to 39.9% [1]. Colorectal cancer (CRC) is the third most frequent cancer type (10% of cancer diagnoses) and the second leading cause of cancer-related death, while gastric cancer (GC) ranks among the top three cancers in 19 countries [2,3]. In 2022, the incidence of CRC (reported as new cases per 100,000 people) was 18.4 worldwide, with the lowest rate of 5.5 in Middle Africa and the highest rate of 35.33 in Australia-New Zealand [4]. GC incidence appears to be lower, with the highest rate of 30/100,000 reported in East Asia in 2020 [2]. Moreover, global trend predictions are not optimistic. CRC incidence is expected to increase by 70.53% by 2045, including a peak increase of 129.64% in East Africa [4]. GC incidence is projected to increase by 62% by 2040 [2].

### 1.2. Tumor Cells and Neighboring Microenvironment

The tumor microenvironment (TME) is described as an extensive, diverse and dynamic ecosystem that continuously interacts with tumor cells which arise, survive and progress within it [5]. From a historical perspective, the first descriptions of TME cells assigned them a bystander role. However, later experiments and observations described key functions in tumor progression, immunosuppression, and therapy resistance [5,6]. TME can be divided into numerous components. The first and most representative are TCs, which constitute between 5% and 100% of the TME volume [7]. Immune cells are located in the vicinity of TCs and constitute the inflammatory tumor microenvironment (iTME), with some of them having a significant impact on TP. The other cell populations identified within the TME are non-tumor, non-immune cells: endothelial cells, pericytes, fibroblasts, adipocytes, mesenchymal stem cells, and neuronal cells. Within the TME, molecules such as cytokines, involved in paracrine interactions, and extracellular matrix components, are also identified [5,6,8,9,10]. Another characteristic of the TME is hypoxia [6]. Higher hypoxia-related scores and molecular signatures are associated with shorter overall survival (OS) [11,12]. Numerous and complex interactions between TCs, tumor-associated macrophages (TAMs), and T lymphocytes have been described within the TME, and these regulate TP in a symbiotic manner. Throughout this review, molecular or histological subtypes are specified where reported in the primary studies. When subtype information is unavailable, the findings should not be interpreted as uniformly applicable across all CRC or GC subtypes.

### 1.3. Current Treatment Strategies and Their Outcomes

Although colonoscopy screening has decreased CRC incidence, mortality has not been affected [13]. As a treatment option, surgery remains an important part of digestive cancer management, and 5-year overall survival could increase to 84.1% [14]. Standard chemotherapy protocols (alone or complementary to surgical resection) are combined with various molecules that target intracellular pathways or surface receptors of tumor cells, with improved outcomes being observed. As an example, a phase III clinical trial has reported twofold longer OS in the mFOLFOX6 + anti-EGFR + anti-BRAF arm compared with the standard-of-care arm (NCT04607421 [15]). Immunotherapy is beginning to show advantages over conventional therapies in clinical trials, as Wang et al. noted in their meta-analysis [16]. However, the projection of cancer-related deaths for 2045 shows an increase of 83.47% compared with 2022 [4]. Moreover, 1.27 million deaths among GC patients are expected by 2040 [2].

All of the above shows the promising potential of immunotherapy and raises considerable interest in the manipulation of various interaction mechanisms between tumor cells and immune cells in order to improve the outcomes of patients with gastrointestinal cancers.

### 1.4. Scope of This Review

The importance of studying these interactions stems from the epidemiological data on malignant digestive cancers and the current outcomes of widely used treatments. Despite the fact that the TME is a highly complex system with a variety of interactions between its components, we aim to focus in this review on those recently described in the literature: (i) ligand-receptor or paracrine signaling between tumor cells and CD8+ T-cells and the intracellular pathways involved, (ii) ligand-receptor or paracrine signaling between tumor cells and macrophages and the intracellular pathways involved, and (iii) current treatment strategy outcomes and clinical trials based on the mechanisms described in the first two points. This review and its search strategy are based on two questions: “What has been discovered in recent years about these interactions and their impact on tumor progression?” and “What is the current state of therapies targeting these mechanisms?”

## 2. Materials and Methods

### 2.1. Databases and Search Strategy

For this literature review, the PubMed and Web of Science databases were analyzed and were last accessed on 8 July 2026. The search strategy was a general-to-specific one, starting with search terms such as: “Colorectal cancer tumor cell and macrophage”, “Gastric cancer tumor cell and macrophage”, “Colorectal cancer tumor cell and CD8+ T lymphocyte”, and “Gastric cancer tumor cell and CD8+ T lymphocyte”, grouped yearly within the 2020–2026 time frame. The next step was to use key molecules/mechanisms identified in the previous step as a new foundation for the search strategy (e.g., “Galectin-9 and colorectal cancer and CD8+ T-cell exhaustion”) to find results that supported or contradicted the previous ones. Duplicates were manually identified and removed. At the end of the search period, an updated search was conducted to identify studies published during the time elapsed since the initial search. Depending on the search terms used, the number of retrieved records ranged from 3 (“colorectal cancer” and “macrophages” and “ADAR1”) to 2578 (“colorectal cancer tumor cell” and “macrophages”). During study-selection process, A.P. screened titles and abstracts according to inclusion and exclusion criteria. Further, the full texts of potentially eligible studies or studies for which eligibility could not be determined from the abstract alone were assessed to reach a final inclusion decision. The final selection of the included studies was subsequently cross-checked by V.I. and M.D.

### 2.2. Inclusion and Exclusion Criteria

To meet the inclusion criteria, a study had to focus on molecular mechanisms that govern tumor cell-CD8+ T lymphocyte/macrophage interactions within the microenvironment of digestive malignancies. Only studies published in English and available in full text were included. For this purpose, research articles that were published within the mentioned time frame and primarily focused on the mechanism of interest were selected. To answer the review questions, studies using various methodological approaches were included, based to some extent on online biodatabases, cell lines, and animal or human tissue-based methods (see Section 6). Secondarily, 32 studies published before the 2020–2026 time frame were included for their enduring value and the relevance of the information they provided. Among these studies, 11 were included in Section 6, “Current therapeutic strategies and clinical trials,” and 21 landmark references were scattered throughout the rest of the text. For Section 6, conference abstracts reporting results from the included clinical trials were also considered when these results were not available in full-length publications at the time of the search. Twenty-five studies focusing on non-tumor subjects were included for their highly valuable information about immune cell response. Knowledge gaps such as incompletely described mechanisms for gastric or colorectal cancers, unknown related mechanisms, or controversies were addressed by including 22 articles focusing on non-digestive tumors, which provided additional support.

Studies were excluded if they did not address this review’s questions or mechanisms of interest. Studies investigating non-digestive malignancies or published before 2020 were excluded from the primary analysis. However, some studies focusing on other cancers were considered to provide complementary mechanistic evidence relevant to this review. Of particular importance, this review did not include articles that mentioned mechanisms of interest as a matter of fact, without further contextualization, or articles without full-text access.

## 3. Tumor Cell–Lymphocyte Interactions

CD8+ T-cells are a highly effective component of the immune system involved in tumor cell eradication, and they represent a priority target of immunotherapy [17,18] because of their recognition and elimination capabilities through intracellular signals and pathways mediated by MHC I molecules.

Physiologically, when CD8+ T-cells are stimulated by an antigen and receive co-stimulatory signals, they enter a proliferation phase and differentiate into effector or memory CD8+ T lymphocytes. If these CD8+ T-cells are exposed to continuous stimuli, inflammatory or tumoral in nature, molecular and functional changes may develop, driving CD8+ cells to a dysfunctional state [18]. Lymphocytes continuously communicate with TCs, and the number of CD8+ T lymphocytes is highly correlated with tumor progression [19]. The density and functional competence of T lymphocytes within the TME represent crucial determinants of immunotherapy success and an efficient antitumor immune response.

The number of T-cells in CRC is lower compared to normal mucosa [19], with the number and density of T-cells decreasing in late stages [20,21,22]. The density of T-cells in the tumor center and invasive front is highly variable; some authors have observed that right-sided CRCs feature fewer T-cells [20], with the invasive front presenting a higher density compared to the tumor center [21], while others suggest that T-cells are frequently excluded from close proximity to tumor cells at the tumor-stroma interface, in contrast with macrophages, and exhibit a lower immune response mediated by decreased cytokine production [23].

Lymphocyte populations are characterized by substantial heterogeneity within the TME; two examples from GC are CD8+ T-cells expressing CD226, associated with better survival [24], or CD39, characterized by an exhausted state [25]. A rare subset comprises T γδ cells, further divided into Vδ1, Vδ2, and Vδ3 subtypes, with the first two showing an association with a cytotoxic effect but being rarely identified within the CRC TME [26]. The CD8+ T-cell compartment comprises functionally active cells, precursor exhausted CD8+ T-cells (Tpex), and exhausted CD8+ T-cells (Tex), with their relative proportions changing dynamically [27]. This ratio was estimated and named PETER (Progenitor Exhaustion-to-Terminal Exhaustion Ratio); low values, corresponding to a higher Tex cell count, indicated a worse immunotherapy response [28].

Early stages of CRC show a significant density of the cytotoxic T-cell population transitioning to an exhausted phenotype with tumor progression [20]. In particular, Tex are observed in tumor tissue but not within non-tumor colorectal mucosa [19]. In contrast, Tpex cells can be identified in both tumor and non-tumor tissue [24]. The pathological mechanisms of exhaustion are multiple and incompletely understood. Some evidence indicates prolonged antigen exposure as a predominant factor because the TCR is continuously engaged and initiates the exhaustion process [29]. Other findings suggest strong communication between Tex and TCs through CD99-CD99, Migration Inhibitory Factor (MIF)-(CD74-CXCR4), or MIF-(CD74-CD44) with possible roles in the exhaustion process [19].

Lymphocytes identified as terminally exhausted exhibit reduced function, characterized by a low proliferative rate, cytokine synthesis and secretion and, more importantly for antitumor function, low cytolytic activity [27]. Throughout this process, CD8+ T-cells can be found in an intermediate state referred to asTpex [18] or as a progenitor exhausted by other authors [30]. CD8+ T-cells can be differentiated into stages of exhaustion based on molecular expression patterns. Tpex cells are characterized by the expression of TCF1, PD-1, CD8, activation markers (CD69, CD107), inhibitory immune checkpoint molecules (LAG3, CD200) and transcription factors (ID3, EOMES, T-bet) [17,18,31]. These cells share common characteristics with early memory T lymphocyte surface markers such as CCR7, IL7R and CD26L [27].

Tex lymphocytes are characterized by the expression of CD39, CD244 and TIM-3 [27]. Transcriptional changes can also be identified, with TOX and NR4A being highly expressed [17,32,33], as well as epigenetic changes manifested as a decrease in chromatin accessibility for some effector genes and an increase in chromatin accessibility for some exhaustion-associated genes [33].

Favorable results of anti-PD-1 therapy are immunologically dependent on CD8+ T-cell function, reestablishment of cytolytic function and the capacity to counter the immunosuppressive effect of the TME [17,18], with a higher number of CD8+TCF1+ (Tpex) cells being associated with better therapeutic outcomes. One highly significant fact is that Tpex lymphocyte proliferation can be induced, with these cells being able to repopulate and maintain the T-cell response against tumor cells. This capacity is observed among TCF1+ T-cells but not among TCF1- T-cells (Tex) [31]. As a result, one hypothesis is that the low number of patients responding to anti-PD-1 therapy can be explained by the inability to activate dysfunctional Tex cells [18].

In the TME, a lymphocyte subset called “bystander T cells” can be identified, comprising non-specifically activated lymphocytes that happen to be present during the development of the immune response [27,34]. These bystander lymphocytes express a variety of markers such as memory T-cell markers (CCR7, CD27, CD28) and exhaustion markers (PD-1, TIGIT). This particular situation makes the distinction between Tpex and Tex difficult because of their shared molecular characteristics [27]. CD83 is a molecule that can differentiate between these two cell subpopulations because T lymphocytes express CD83 only after antigen stimulation and its expression decreases with the development of the Tex state. A marker that can differentiate bystander lymphocytes from Tpex lymphocytes is highly useful because of the common marker expression of these cells [27].

The interaction between T-cells and TCs is not the only determinant. Various cells that form the iTME and have immunosuppressive effects, such as tumor-associated fibroblasts (TAFs), tumor-associated macrophages, myeloid-derived suppressor cells (MDSCs) or regulatory T-cells (Tregs), can initiate and facilitate the exhaustion process [35].

Another crucial perspective is the metabolic one, dominated by hypoxia, which determines mitochondrial dysfunction and stimulates reactive oxygen species (ROS) synthesis, leading to T-cell exhaustion [32,33,36]. Detailed analysis of the influence of hypoxia on the immune cell populations within the TME revealed an increase in inhibitory subsets (including macrophages) and a decrease in stimulatory subsets (for example, central memory T-cells) [36]. The cumulative effect is a significant number of inflammatory cells with a reduced cytotoxic capacity, with hypoxia-related pathways showing the greatest upregulation in CMS4 CRC subtype [37]. Hypoxia directly influences the antitumor response via HIF-1α expression in TCs associated with follistatin-like 3 (FSTL3) overexpression [38], which favors an increase in PD-L1 expression by TCs [39] and epithelial-mesenchymal transition (EMT) through various pathways [40,41]. FSTL3 expression is higher in MSI-H CRC tumors [39]. Nutrient competition and metabolite accumulation, including lactate, in TME can initiate or sustain T-cell exhaustion [35].

The PI3K/AKT pathway represents one of the most important and widely studied signaling pathways in cancer and is involved in proliferation, apoptosis, neoangiogenesis, autophagy, EMT and chemoresistance [42]. The first step is PI3K activation and the formation of phosphatidylinositol 3,4,5-trisphosphate (PIP3), which further activates AKT [43]. The biological functions of this pathway highlight its crucial role in tumor initiation and progression [44,45] and treatment resistance [46,47].

In TCs, this axis is stimulated by various molecules (Figure 1), for example, HECW1—overexpressed in GC [48] and mediated by HIPK2 [49] or YWHAH, a signaling and intercellular interaction protein—via FRA1/HMGA1 stimulation [50]. A TME with overexpression of these molecules is characterized by few Tex cells and a high density of TAMs as a result of Hippo pathway and IFN-α/TNF-α downregulation [51] and increased expression of PD-1/TIM-3 on TCs. In addition, YWHAH also activates the MAPK/ERK pathway. Its precise mechanism of direct interaction with lymphocytes is unknown. Although YWHAH was associated with MSI status, based on a TCGA-TIDE score, clinical MSI-H/MSS status was not reported [52].

This pathway can be activated at the AKT level by FSTL3, a glycoprotein detected in numerous cells and related to cell growth, metabolism and various cancers, including CRC [39,53]. Cancer-associated fibroblast-derived FSTL3 binds to TfR1 on TCs and activates the AKT/mTOR pathway [54]. This molecule has multiple targets, activating c-Myc to increase PD-L1 levels [39] or HIF-1α [38,55], and its expression is reduced by ZNF454 [55]. Apart from the FSTL3-MSI association described above [39], molecular subtype information was not reported in these mechanistic studies, limiting the assessment of subtype-dependent effects.

The PI3K/AKT pathway can be suppressed within CD8+ T-cells as a result of crosstalk with CD155-expressing TCs. CD155 is a receptor first described as the poliovirus receptor and now frequently identified on malignant TCs [56,57,58], with known inhibitory effects on immune cells through crosstalk with various surface molecules [59]. A highly CD155-expressing TME is immunosuppressed [60] and characterized by few Tex cells, thereby indicating a poor prognosis associated with the CD155-TIGIT axis [56,61]. The CD155 level is also upregulated by CEBPB in GC cells [62].

This molecule interacts with TIGIT [57,63], whose expression is higher in MSI and BRAF-mutant CRC [60], located on the surface of CD8+ T-cells, intracellularly inhibiting the PI3K/AKT pathway and subsequently NF-kB [56], but other pathways can be involved, such as mTOR in triple-negative breast cancer [63], although these have still not been researched or described in digestive malignancies. In contrast to previous results, He et al. reported an increased level of AKT phosphorylation in GC, while CD155 was downregulated [64].

The activity of the PI3K/AKT and ERK pathways is downregulated in CD8+ T-cells by the KLRB1 receptor as a result of binding to CLEC2B, a ligand identified on the membrane of CRC TCs, as demonstrated primarily in MSS CRC models [65]. Some results indicate increased cytotoxic activity of CD8+ T-cells and low PD-1 levels as a result of PI3K/AKT/mTOR axis activation by interferon-induced protein 35 (IFI35), highly expressed in CMS1 CRC and secreted by TCs in response to IFN-γ exposure [66].

The PD-1 level in TCs is positively influenced by lipin 3 (LPIN3) and YTH N6-methyladenosine RNA-binding protein 1 (YTHDF1). LPIN3, whose expression correlates with MSI in TCGA data, plays physiological roles in lipid metabolism but also promotes TP in CRC via β-catenin activation. Its inhibition raises IFN-γ levels, enhancing the cytotoxic CD8+ T-cell response [67]. However, prolonged exposure to IFN-γ could initiate T-cell exhaustion [68].

Similar to LPIN3, YTHDF1 is known for its involvement in TP and metabolism. It is highly expressed by GC TCs, with lower expression in the genomically stable (GS) subtype, and correlates with the mutant p53 state, a low number of inflammatory cells and their exhausted state [69], and the risk of peritoneal metastasis [70]. The changes noticed after YTHDF1 knockdown reveal a reshaped antitumor TME [71,72].

Moreover, the PD-1 level is increased in CD8+ T-cells as part of the PKA/CREB pathway activity, with a similar tendency being observed for TIM-3 levels as a result of thyroid-stimulating hormone receptor (TSHR) activation. TSHR is highly expressed in tumor and stromal cells of CRC as well as in CD8+ T-cells. TCs employ extracellular vesicles (EVs) to deliver TSHR to lymphocytes. Its high expression is associated with MSS status and immune checkpoint blockade failure [73]. A similar mechanism has not yet been identified in GC.

Intracellular domains of PD-1 and TIM-3, two well-known molecules with inhibitory effects in digestive malignancies [74,75], are crosslinked by Galectin-9 [76], a β-galactoside-binding lectin identified in numerous immune cells and associated with physiological roles such as cell growth or apoptosis [77]. In GC, galectin-9 is associated with TP [77], and in CRC, the TIM-3/Gal-9 axis supports an immunosuppressive TME [78,79] characterized by high LAG3 and TGF-β levels with a known inhibitory impact on CD8+ T-cell function [79]. TIM-3is also expressed by various immune cells, with its level in CD8+ T-cells being correlated with a better prognosis when it is coexpressed with PD-1 [80], and M2-like macrophages raise TIM-3 levels as a result of TGF-β stimulation. This axis is reported in MSI, CMS1 and CMS4 TME [81]. Some authors consider Galectin-9 a ligand for the TIM-3 receptor with protumoral effects [82]. However, TIM-3 inhibition reveals better outcomes of anti-PD-L1 treatment in predominantly pMMR experimental settings [78].

Another mode of interaction between TCs and CD8+ T-cells is mediated by various cytokines, such as IFN-α [83], TGF-β [30], IL-10, IL-6, IL-35 or IL-27, that initiate and sustain the T-cell dysfunction process [32].

TGF-β is secreted by various T-cell subtypes [26,84] and even by CRC TCs [85], with known roles in CD8+ T-cell exhaustion [86]; increased infiltration of cytotoxic CD8+ T-cells has been observed as a result of TGF-β and PD-L1 co-inhibition in a CMS4-like mouse model [87]. TGF-β has various roles within TCs, with the TGF-β/Smad axis being activated by multiple factors: Forkhead box protein C1 (FOXC1) [88], ADAM17 [89], SNHG12 [85], Prkci [90] or ARHGAP4. This pathway stimulates colorectal tumor cell proliferation, migration, epithelial-mesenchymal transition and invasion [91].

One essential cytokine in the immune response is IL-6 [92]. Within the TME, it can be released by CD3+ cells [93] or TCs [85,94,95] and promotes TP via STAT3 activation [93,96], either directly, by VEGF upregulation [96], or as a promoter of the metastatic process [97]. The IL6/JAK/STAT3 pathway is also activated by HOXC6, which is highly expressed in MSI-H CRC TCs [98]. In contrast, Pennel et al. observed that high stromal IL-6 expression is associated with poor prognosis but not with its expression in TCs [95]. The level of IL-6 modulates immune populations, with its overexpression being correlated with an immunosuppressed TME. ASCL2 is one key factor that directly influences IL-6 levels and, consequently, T-cell numbers, as described primarily in MSS CRC [99]. Other cytokines, such as IL-12, increase the numbers of Tpex cells and M1-like macrophages [100], and IL-2 stimulates TME infiltration by M2-like macrophages and CD8+ T-cells [92].

CRC TCs frequently exhibit urocortin (UCN), a member of the corticotropin-releasing hormone family involved in inflammation and tumorigenesis. UCN facilitates TP by CCL23 upregulation, a T-cell exhaustion-promoting chemokine [101], or by p53 inhibition by UCN-1 [102]. Increased levels of T-cell exhaustion markers induced by CCL23 in non-digestive cancers were reported as part of the effect of GSK3β activation [103], a substrate of the PI3K/AKT pathway [104], with no evidence of the CCL23/GSK3β axis being involved in TP in digestive cancers.

Major facilitator superfamily domain-containing 2 (MFSD2A) is a molecule identified in multiple tissues with various actions (TP, hepatic regeneration) [105], with its expression decreasing from normal mucosa to tumor tissue [106,107]. MFSD2A overexpression reduces TAM abundance and promotes T-cell infiltration and function through COX2-mediated reduction in TGF-β1. The results are based on a human GC cohort predominantly MSS/MSI-L (96,3%), EBV-negative (59,3%) [108]. These results are consistent with findings showing the antitumor effect of MFSD2A in CRC via the activation of S100A14 and secondary inhibition of STAT3 [106], or inhibition of the ERK/MAPK pathway with a secondary decrease in PD-L1 levels and a better cytotoxic T-cell response [109].

The desired effect of the interaction between a CD8+ T-cell and a TC is the programmed cell death of the latter—for example, ferroptosis or pyroptosis—with a mechanism based on glutathione peroxidase 4 (GPX4) and GPX8. Their overexpression is observed in multiple cancers, including GC, where it correlates with prognosis and the extent of immune cell infiltration [110,111]. GPX4 activity is based on SLC7A11, but conversely, its overexpression results in decreased ferroptosis [112] and high TRIB3 expression [113]. A TME with SLC7A11 overexpression presents low CXCR3 and CXCL14 levels [112]. CXCR3 does not modulate CD8+ T-cell numbers [114], but CXCL14 can stimulate the infiltration of immune cells and antitumor action [115,116]. As part of the pyroptosis mechanism, DFNA5 (GSDME) exhibits low expression in Epstein–Barr virus-associated GC [117] and is correlated with exhaustion markers and the number of M2-like macrophages [118].

## 4. Tumor Cell–Macrophage Interactions

Complex and reciprocal interactions between TCs and TAMs have been described that lead to immunosuppression, chemoresistance and TP, with the density of the latter being directly linked to prognosis [119,120]. Macrophages represent the most frequently identified cells in the iTME, accounting for up to 40% of immune cells [17,28,121]. Right-sided CRCs tend to exhibit an anti-inflammatory SPP1+ phenotype of TAMs [20] that is associated with a worse prognosis [122,123,124]. However, regardless of side, macrophages tend to start with a pro-inflammatory state and end with an anti-inflammatory one [20].

Macrophage activation has traditionally been described using an M1/M2 polarization framework that plays major roles within the TME. The M1/M2 framework developed from earlier studies demonstrating differential regulation of macrophage nitric oxide synthase and arginase activity by Th1/Th2 cytokines [125] and was subsequently popularized by Mills et al. in 2000 [126].

However, this binary classification represents a simplified representation of a broader and highly plastic spectrum of macrophage activation states, a complexity further revealed by single-cell RNA sequencing, which has identified macrophage phenotypes such as SPP1+, C1QC+, INHBA+, ISG15+, LNRP3+ or LYVE1+, the first two being well known for their influence on the immune response within the TME [127].

Spatially, macrophages tend to be located at the TC-stromal interface, at the invasive front [128], where they assemble a barrier-like structure that promotes local immunosuppression. Moreover, macrophages are located closer to TCs than T lymphocytes [23]. SPP1+ macrophages, a specific subtype, are identified in digestive malignancies in the following order: tumor core > invasive margins > non-tumor mucosa [20,129,130,131,132]. In contrast, one study identified the highest density of SPP1+ macrophages at the invasive front [133]. Moreover, the number of SPP1+ macrophages is higher in metastases compared with the primary tumor [124,129]. The SPP1+ TAM signature is enriched in MSI-H CRC TME [129].

Osteopontin, present in intracellular or extracellular secreted forms [120], is the glycophosphoprotein encoded by the SPP1 gene and fulfills various roles in the TME, showing a significant impact on M2-like macrophage polarization (Figure 2) [134]. SPP1 is associated with a negative prognosis because of its pro-metastatic activity in digestive malignancies and the co-synthesis of PD-1, PD-L1, VEGF and MMP2, collectively promoting TP [129,131,135,136]. However, SPP1+ TCs do not correlate with migratory or invasive capacity [134]. Taken together, these findings support SPP1+ macrophages as relevant biomarkers of TP. At the opposite end of the spectrum are CICQC+ macrophages that exert cytophagic and MHC-II antigen-presenting capacities [129,136].

Numerous interactions between TCs and macrophages center on osteopontin. TCs can promote the SPP1+ phenotype of macrophages through intracellular RING finger 32 (RNF32), which activates the Wnt/β-catenin pathway, firstly promoting TP and secondly increasing CCL2 levels. This chemokine interacts with the CCR2 surface receptor on macrophages and promotes SPP1 expression [137]. Another molecule delivered by TCs is migration inhibitory factor (MIF), which binds to the CD44 receptor. The subsequently activated pathways lead to BCL9L stimulation and the SPP1+ phenotype [138].

Hypoxia plays an important role in the development of the SPP1+ state, which is enriched in CMS4 tumors [139], with these cells exhibiting hypoxia-associated [129] or angiogenesis-associated genes, such as VCAN [140], leading to increased angiogenic capacity of SPP1+ macrophages [124,136], and with a high number of these cells within the TME being correlated with lymphovascular tumor invasion [123,136]. High levels of HIF-1α, increased by hypoxia, interact with STAT3 and, finally, promote the development of the SPP1+ phenotype [135]. TC-derived osteopontin influences macrophages by upregulating PI3K/AKT within these immune cells and increasing CSF1 levels, a known factor for macrophage recruitment and the promotion of the M2-like phenotype [134].

Osteopontin binds to the CD44 receptor located on the surface membrane of TCs, thereby activating the Wnt/BTF3 pathway and promoting TP [141,142]. Moreover, SPP1+ macrophages activate JAK/STAT3 in MSS CRC TCs [143]. In diffuse-type GC, SPP1+ macrophages are promoters of TP, a worse prognosis and macrophage recruitment [131]. Osteopontin interacts with TCs directly through αvβ3 integrin [135] or indirectly through PDGFB upregulation, which, as a result of binding to a TC receptor and crosstalk with FADS1, activates the mTOR pathway [144].

Collectively, these observations describe TP as a result of direct interactions between SPP1+ macrophages and TCs. However, TP can also occur as a result of multiple interactions with CD8+ T-cells and their exhaustion. TAMs possess a high phagocytic capacity and the ability to process tumor antigens; however, they are unable to initiate or sustain the T-cell activation process [17].

SPP1+ TAMs interact directly with PD-1+ CD8+ T lymphocytes, an interaction mediated by SLC2A1 [145], a molecule upregulated by YAP1—a promoter of invasive behavior through the Wnt/β-catenin pathway [146]. Another result is the downregulation of the LCK-Zap70-LAT axis that transfers the TCR signal to the nucleus [132]. This macrophage subset produces CXCL16, CXCL9, and IL-10, which may influence CD8+ T-cell activity and trafficking. Collectively, these findings suggest that macrophages may contribute substantially to T-cell exhaustion.

Initially characterized for its role in metabolism and phagocytosis, studied frequently in neuropathology [147], and reported to have anti-inflammatory roles in non-alcoholic liver steatosis [148], triggering receptor expressed on myeloid cells 2 (TREM2)—a type I transmembrane immunoglobulin receptor expressed by macrophages [149,150]—is now recognized for its role in multiple cancers [151]. Upon ligand binding, signaling through the adaptor proteins DAP10 and DAP12 leads to the activation of the PI3K/AKT pathway [150]. TREM2 expression is identified in TCs and macrophages, with both digestive malignancies showing its overexpression [151,152,153]. In GC, TREM2 expression was inversely associated with HER2 expression, with no significant differences being observed between histological types [153]. In TCs, it acts as a Wnt/β-catenin/TCF4 pathway inhibitor, decreasing TP [154], with a high impact on 5FU treatment resistance in GC when it is underexpressed [155]. TREM2 knockdown significantly reduced the proliferative capacity of TCs and their invasion ability and increased E-cadherin expression levels, with a known role in EMT [152]. In lung adenocarcinoma, the transfer of circ_0001715 from TCs to macrophages via extracellular vesicles was observed to increase TREM2 levels and facilitate TP [156]. At present, there is no evidence in the literature of such a mechanism occurring in digestive malignancies.

TREM2 activity within TAMs is highly correlated with STAT1 phosphorylation, leading to high CCL8 and PD-L1 levels [157], which accentuate CD8+ T-cell exhaustion. Moreover, the TREM2+ signature was associated with the EBV molecular subtype of GC [158]. As expected, anti-PD-L1 treatment outcomes can be improved by targeting this molecule [157]. A hypothetical interaction with APOC1 exhibited by TAMs can elevate CCL2/5 levels within the TME [159,160]. TREM2+ macrophage density can increase under glucocorticoid exposure [161].

The Wnt/β-catenin signaling pathway is well known for its impact on the progression of digestive malignancies [162,163], with macrophages activating this pathway in TCs by TGF-β secretion and using tribbles pseudokinase 3 (TRIB3) as a mediator [164]. TRIB3 is known to function as a key factor in physiological cellular proliferation, differentiation and also TP, being overexpressed in CRC [113,165,166].

In TCs, HIF-1α upregulates TRIB3 [164,167], which interacts with COP1 to increase β-catenin levels [167].

Another pathway influenced by TRIB3 is centered on STAT3. Furthermore, this enhances VEGFA expression and supports TP through neoangiogenesis [165]. Another promoter of the TRIB3/VEGFA axis in digestive malignancies is TEAD4 [113,168], with its overexpression being correlated with high ferroptosis resistance in TCs through activation of the MEK/ERK axis and increased GPX4 and SLC7A11 levels [113,169]. An inhibitor of this pathway is METTL14 [169].

Without strong evidence, TRIB3 could activate the PI3K/AKT pathway under high-glucose conditions in diabetic patients [170]. In contrast to previous studies, Liu et al. identified opposite results characterized by a decrease in TRIB3 in GC with TP. Notably, a TME with high TRIB3 expression is associated with an increased number of M1-like macrophages and better survival [171]. As a member of the same family, TRIB2 plays a similar tumor-suppressor role in GC [166].

The Wnt/β-catenin axis is also stimulated in TCs by ankyrin repeat domain-containing protein 22 (ANKRD22), physiologically identified in normal gastric epithelium and activated macrophages but overexpressed in CRC TCs [172,173]. Furthermore, FOSL1 gene transcription is promoted [173], neoangiogenesis is increased by FOSL1/TIMP1/VEGF [174], and TCs become more resistant to 5FU [175]. There is a limited number of studies in the literature on ANKRD22 and intercellular interactions in digestive malignancies. Similar tendencies in TP are described for other tumors such as lung adenocarcinoma [176,177], breast cancer [178] and papillary thyroid carcinoma [179]. In contrast to previous studies, some authors reported an M1-like-promoting effect of ANKRD22 in CRC [180], with similar tendencies in sepsis-induced ARDS [181] or gastric mucosal injury, in which ANKRD22 knockdown decreased M1-like polarization without an impact on M2-like macrophages [182].

Macrophages interact with and influence TCs through the secretion of matrix metalloproteinases (MMPs), members of the metzincin family with a well-established role in extracellular matrix proteolysis, with a subset of them (MMP1, 3, 7, 9–12, 14) being markedly increased in CRC [183]. MMP1 is expressed by TCs, T lymphocytes and TAMs [184], and MMP1+ TCs represent a pre-metastatic population [184]. Tumor cells stimulate MMP1 secretion by macrophages, mediated by GM-CSF as a result of stimulation of the PI3K/AKT pathway in these immune cells [185]. Conversely, M2-like macrophages can promote TC invasion through cytokine synthesis, such as TNF-α, which stimulates MMP9 secretion by TCs [186], or TGF-β, which facilitates MMP2 secretion [187].

MMP1 secreted by TAMs binds to the TC receptor PAR1, which favors EMT via the MAPK/Erk axis [188,189,190]. This receptor may contribute to TP via the 3-phosphoinositide-dependent kinase 1 (PDK1)/AKT pathway in KRAS mutant CRC [189]. TP is also positively influenced by MMP1 expression in TCs as a result of its intra- and/or extracellular roles. MMP1 synthesis by TCs is enhanced by numerous factors: CD26/CAV1 axis activation [191], HIF-1α [192], zinc finger protein 3 (ZNF3) [193] or calmodulin-regulated spectrin-associated protein 2 (CAMSAP2) [194]. In contrast to these results, Ito et al. reported decreased TC proliferation in line with higher MMP1 expression in GC TCs [192]. From another perspective, MMP1 interacts with and exhausts CD8+ T-cells through the CXCL16-CXCR6 axis [184]. Tissue inhibitor of metalloproteinase-2 (TIMP2) reduces the proteolytic capacity of metalloproteinases (particularly MMP2). TIMP2 has certain roles in digestive malignancies but not as prominent as in other cancers. mRNA levels reveal decreased expression in CRC but increased in GC, subtypes not reported in a primary study. TIMP2 expression in both digestive cancers correlates with TAMs, the number of CD8+ T-cells and their exhaustion status within the TME [195].

On the other hand, TCs can influence macrophage behavior using various molecules (Figure 3) such as ADAR1, an RNA-editing enzyme highly expressed in CRC [196] with known roles in TP [197]. TCs use extracellular vesicles to transfer this molecule to macrophages [196] through four known mechanisms: increasing AZIN1 levels to promote an M2-like phenotype [196], activating the PKR/EIF2α pathway to decrease TIM-3 levels, with a well-known impact on T-cell cytotoxic function [198], favoring SPP1 secretion, which activates the NF-*κ*B axis in TCs to increase resistance to oxaliplatin treatment [196], or activating the Wnt/β-catenin/CALR pathway (in gastric cancer cells) [199]. The pro-M2-like effect of ADAR1 is also reported in non-tumor diseases [200].

TCs secrete chemokines belonging to the CC or CXC families, with minimal structural differences [201], to influence macrophage behavior. CXC motif chemokine receptor 2 (CXCR2) is a chemokine receptor identified on several immune cells that interacts with seven ligands differentially expressed by various tumors. Stimulation of this receptor on macrophages leads to activation of the PI3K pathway. CRC TCs secrete CXCL1–3, 5, 8, while GC TCs secrete CXCL1, 3, 5, 6, 8 [202], with a subset of them (CXCL3, 4, 14) being correlated with macrophage numbers within the TME [203]. CXCL1 is overexpressed by CRC TCs [204]; however, the mechanism can be bidirectional, as macrophages secrete CXCL1 within the TME and promote TC invasion [205]. CXCL1 and CXCL3 are overexpressed in CRC TCs as a consequence of the activation of the NF-*κ*B pathway by SUB1 [206]. CXCL8 is a chemokine secreted by CRC TCs that increases TAM numbers and promotes M2-like polarization, with these effects being mediated by STAT3 [207]. A further decrease in the number and function of CD8+ T-cells is modulated by M2-like macrophages [207] or via NETs (neutrophil extracellular traps) that are stimulated by CXCL8 [208,209]. Conversely, Chen et al. reported a positive gradient between CXCL8 and the number of immune cells/cytotoxic T-cells within the TME [203], while Korbecki et al. reported that CXCL8 secretion in CRC is associated with macrophage infiltration without M2 characteristics [202]. TAMs secrete CXCL9/10, which binds to CXCR3 on TCs, promoting EMT, increasing PD-L1 expression and T-cell exhaustion. Macrophages use this mechanism through STAT3 activity and decreased expression of NF-kappa B activator 1 (Act1) [210].

CCL2 (also known as monocyte chemoattractant protein-1) is expressed by TCs in digestive malignancies and has a major impact on macrophage recruitment and M2 phenotype polarization [211,212,213]. Numerous factors that positively modulate its level within TCs are described in the literature: STAT3 [214] (which is stimulated by PIPKIγ [215], ETV5 [214], VSIG4 [216], HMGA2 [213]), GAL3ST4 (in KRAS mutated CRC) [217], TCF4 [218], RUNX1 [219], HOXC6 (in MSI-H CRC) [98], HOXD13 (via TGF-β1-p-Smad in CMS4/MSS CRC [220]) and SLITRK4 (via PI3K/AKT/NF*κ*B) [221]. This chemokine binds to the CCR2 receptor and stimulates multiple intracellular signaling pathways [212], including the Hedgehog pathway in macrophages, to facilitate M2-like polarization [219].

Conversely, M2-like TAMs secrete CCL2 within the TME via the Wnt5a/CaMKII/ERK pathway, enhancing macrophage recruitment [222]. CCL2 levels can be decreased in GC, for example by CDK5RAP3, leading to M1-like macrophage polarization [223]. In addition to CCL2, TCs can promote macrophage recruitment via interleukins such as IL-6 [128] or CSF1, which is directly correlated with GPSM1 expression and T-cell exhaustion; targeting this pathway reduces anti-PD-1 treatment resistance [224].

TCs promote the development of the SPP1+ phenotype in macrophages [130], which are characterized by a genetic profile similar to that of M2-like macrophages [131]. Within the MSI-H CRC TME, their presence is strongly correlated with SPP1 expression [129]. TCs exhibit ITGB1 and CD44 receptors, with the latter being well known for its interaction with SPP1 [131].

Another described mechanism through which TCs induce SPP1+ macrophage activation is centered on extracellular vesicles (EVs) [225]. Extracellular vesicles are particles released by cells, enclosed by a lipid bilayer, that play roles in intercellular communication through various molecules [226,227]. A large number of EVs have been observed and studied to date, with their main characteristics being summarized in Table 1.

Numerous interaction mechanisms between TCs and macrophages within the digestive TME are described in the literature; however, they remain poorly studied to date. These mechanisms can be grouped as promoting the M2 phenotype: ArfGAP with dual PH domains 1 (ADAP1) [254], SerpinE2 [255], TIPE [256], or the M1 phenotype: USP48 (ubiquitin-specific peptidase 48) [257], MCAM (Melanoma Cell Adhesion Molecule) [258], TRIM26 (Tripartite motif-containing protein 26)—through loss of expression [259], or ZFAND2A [260], described in a genetic prognostic score in GC [261]. An uncommon influence is exhibited by myeloid cell leukemia 1 (MCL-1), part of the BCL-2 cluster, which has an anti-apoptotic role and is involved in the pathogenesis of a number of cancers [262]. CRCs with high MCL-1 expression have an increased TME density of M1-like and M2-like macrophages and increased PD-L1 expression [263]. On the other hand, M2-like macrophages highly express AKR1B1, with a currently unknown impact on TCs [264]. High MCL-1 and AKR1B1 expression is identified predominantly in CMS4 and CMS1 subtypes of CRC [263,264].

The main molecular interactions between TCs and macrophages or CD8+ T-cells, along with their key characteristics, are summarized in Table 2.

## 5. Molecular Subtypes and Their Impact on the Tumor Microenvironment

The associations discussed below reflect the molecular or histological context reported in the cited studies and should not be interpreted as evidence that all the described mechanisms are specific to, or uniformly active within, a given subtype.

CRC and GC comprise distinct molecular subtypes with immune and stromal characteristics that are important for understanding tumor–immune interactions. In CRC, the consensus molecular subtypes (CMS) classification distinguishes several subtypes: CMS1-4, according to transcriptomic features [265].

CMS1 CRC (MSI-immune) is characterized by hypermutation and immune activation [265] and shows a TME subtype with both antitumor immunity and inhibitory influence. Tumor cells express high IFI35 that promotes CD8+ T-cell proliferation and cytotoxicity through PI3K/AKT/mTOR signaling [66]. Increased CXCL9/STAT1 expression and stronger Tpex-TAM association highlight coordinated lymphocyte-macrophage responses [18].

CMS2 (canonical) and CMS3 CRC (metabolic), characterized by Wnt/MYC signaling and metabolic dysregulation, respectively [265]. The studies included in this review provide insufficient evidence for these subtypes in order to establish a link with a mechanism.

CMS4 CRC (mesenchymal), associated with TGF-β signaling, stromal infiltration and angiogenesis [265] and the TME shows high TIM-3 expression and M2-like macrophage signatures [81] with a well-known impact on TP and immunosuppression by TIM-3/Gal-9 axis [78,79]. Single-cell and spatial analyses identify a FAP+ fibroblast/SPP1+ macrophage niche associated with immune exclusion [139]. Another crucial characteristic of CMS4 CRC TME is hypoxia [37]. By means of HOXD13, TCs secrete CCL2 [220] and stimulate macrophage recruitment [222] and M2-like polarization [219]. TCs express MCL-1, which promotes a high macrophage density and an increased PD-L1 level [263]. Macrophages exhibit high AKR1B1 levels, with an unknown impact on the TME [264].

MSI-H/dMMR CRC shows high TIGIT expression [60] and TGF-β/TIM-3/macrophage signatures can also be elevated [81]. Another characteristic is a higher level of HOXC6 and FSTL3. FSTL3 binds to TfR1 receptor on TC and activates AKT/mTOR signaling [54], while c-Myc activation increases PD-L1 expression [39]. HOXC6 expression is associated with M2-like macrophage infiltration [98].

MSS/pMMR CRC frequently shows high ASCL2 levels, which promote fibroblast activation and IL-6 secretion, contributing to CD8+ T-cell exclusion [99]. Furthermore, CLEC2B-KLRB1 interaction and PI3K/AKT and ERK subsequent signaling promote T-cell exhaustion [65]. SPP1+ TAM enrichment of TME can be associated with MSI or MSS tumors [129,143], with these suggesting that multiple underlying mechanisms are involved. Although tumor laterality has been investigated in some CRC studies, the evidence reviewed here does not establish its reliability as a surrogate for molecular subtype classification.

In GC, the TCGA classification identifies EBV-positive, MSI, GS and chromosomal instability (CIN) subtypes [266].

EBV-positive GC exhibits high HAVCR2/TIM-3 expression [58]. TREM2 suppresses CD8+ T-cell function through PD-L1 and arginase-1, contributing to an immunosuppressive TME [158]. The YTHDF1-TSC22D1 axis is involved in TP [72]. YTHDF1 findings are context-dependent: one study has reported relatively high expression in EBV-associated tumors [69], whereas experimental EBV infection reduced its expression [72].

MSI GC shows frequent immune infiltration and checkpoint expression [267]. The studies included in this review provide insufficient evidence for these subtypes.

GS GC is characterized by low YTHDF1 expression [69]. The YTHDF1 impact on GC TME was highlighted in EBV-positive tumors.

CIN GC, in comparison with the MSI subtype, shows higher TRIB3 expression but lower TRIB2 expression [166]. The included articles do not provide enough mechanistic information for further characterization.

The studies included in this review did not establish consistent associations between Laurén histological subtypes and specific tumor–immune interaction mechanisms. Several molecules, such as GPX8 [111] or TIMP2 [195], were assessed in relation to histological type, neither showing predominance.

## 6. Current Therapeutic Strategies and Clinical Trials

Recent advances in the treatment of GC and CRC emphasize a multidisciplinary approach and personalized care. Among treatment options, surgery remains an important part of digestive cancer management. The OS of CRC patients treated by surgical resection after 5 years of follow-up ranges from 79.5% to 84.1% (NCT00995202) [14] and drops to 61.1% for pT4bN0M0 cases that undergo multivisceral resection (NCT06115837 [268]).

Currently, conventional chemotherapy protocols (with or without surgical resection) are increasingly combined with targeted agents directed against intracellular signaling pathways or tumor cell surface receptors, resulting in improved clinical outcomes. As an example, FOLFIRI (5-fluorouracil and irinotecan) alone improved OS to 13.6 months, but when combined with bevacizumab or epidermal growth factor receptor inhibitors, it increased OS to 18.3 months [269]. Similar trends were observed with the standard care protocol vs. mFOLFOX6 + encorafenib (BRAF inhibitor) + cetuximab (anti-EGFR antibody): 15.1 vs. 30.3 months (NCT04607421 [15]). The addition of immunotherapy improves OS, and this strategy represents a real hope for future cancer therapy. One trial (NCT02563002) reported twice the progression-free survival (16.5 vs. 8.2 months) in the pembrolizumab group compared with chemotherapy [270], and another (NCT02912559) reported better 3-year OS for atezolizumab (PD-L1 inhibitor) + mFOLFOX6 compared with chemotherapy alone (86.3% vs. 76.2%) in dMMR/MSI CRC patients [271], with these results being supported by a meta-analysis that confirmed better outcomes with PD-1/PD-L1 therapy compared with standard chemotherapy [16]. Similar outcomes were described in a gastric cancer trial (NCT02872116 [272]). pMMR/MSS CRC patients exhibit worse outcomes with immunotherapy, with a median progression-free survival of 9.6 months for untreated patients in one trial [273], while another clinical trial reported a median progression-free survival of 1.5 months for patients previously unsuccessfully treated for metastatic CRC and subsequently treated with checkpoint inhibitors [274]. However, a clinical trial concluded that PD-1 blockade shows similar pathologic response rates in pMMR/MSS and dMMR/MSI CRC patients [275].

Targeting molecules that are part of the complex communication network within the TME can increase the cytotoxic response of CD8+ T-cells to tumor cells and improve patient outcomes. Positive results have been observed in cell culture or animal model experiments. However, these are not equivalent to the complex in vivo microenvironment of patients suffering from gastric or colorectal cancer. Current human clinical trials are in early phases and limited in number, mostly focused on intracellular pathways rather than surface molecules (Table 3). The optimistic aspect is that a large number of experiments are being conducted and new information may be obtained from future research.

## 7. Discussion and Future Perspectives

Intercellular crosstalk within the tumor microenvironment of digestive cancers is highly dynamic, biologically significant and represents a major driver of tumor progression. Tumor cells establish complex interactions with various immune and non-immune cells—with this review focusing on CD8+ T lymphocytes and macrophages—shaping an immunosuppressive niche that supports tumor progression.

Although CD8+ T-cells are the main effectors of antitumor immunity, sustained exposure to the tumor microenvironment gradually induces an exhausted phenotype characterized by impaired cytotoxic function and reduced proliferative capacity. This process is the result of multiple mechanisms, including ligand-receptor interactions (PD-1/PD-L1, CD155/TIGIT or Galectin-9/TIM-3), regulators of intracellular pathways or soluble molecules such as TGF-β, IL-6 or CCL23. These mechanisms are extensively studied and represent promising therapeutic strategies for multiple non-digestive malignancies [296,297,298,299,300].

Concurrently, tumor cells recruit and polarize macrophages toward the M2 phenotype (known for its immunosuppressive influence) through receptor-mediated interactions, cytokines, chemokines or EV-derived molecules. These interactions have already been described and studied as potential therapeutic targets in various cancers [301,302,303].

This review includes research studies on recently described mechanisms involved in tumor cell–immune cell interactions within the tumor microenvironment of digestive malignancies. These experiments are based on preclinical methods or use human tumor tissue that highlight tumor microenvironment characteristics at a single time point during tumor progression, rather than dynamic changes resulting from tumor progression or a therapeutic intervention. The methodological aspects of the original studies included in this review are summarized in Table 4. It should be noted that reviews or original articles used to provide general information (e.g., “The density of T cells in the tumor center and invasive front is highly variable…”), studies of non-digestive malignancies or treatment-based studies were not included in this methodological comparison.

A total of 132 CRC and 45 GC original articles were included in Table 4 for comparison. The larger number of CRC studies included in our methodological analysis compared with GC studies highlights an imbalance in the available evidence. Several mechanisms are extensively characterized in CRC and their applicability to GC should not be assumed without direct validation. This imbalance also highlights opportunities for future research to determine whether mechanisms characterized in CRC operate in GC and to identify GC-specific pathways of tumor–immune crosstalk.

Around half of each cancer group used online biodatabases (e.g., Gene Expression Omnibus—GEO; The Cancer Genome Atlas—TCGA) to identify molecules or mechanisms involved in the described crosstalk, with further in vivo/in vitro experiments. Cell lines were used in 73% of studies, with HCTs (human colon cancer cell lines) and MKNs (human gastric adenocarcinoma cell lines) being the most frequently used. Experiments based on cell lines were grouped into multiple categories based on their main purpose. The molecular analysis category includes experiments such as RT-qPCR, immunofluorescence (IF), Western Blot (WB) and others, with most (up to 68.9%) cell line-based experiments using such techniques. Genetic engineering, the second most commonly used approach, refers to transfection or gene knockdown-based experiments. Cellular functional assays were used in almost half of the studies and included assays based on cell viability, apoptosis, proliferation, migration or invasive capacity. No more than 9% of studies used co-cultures of human cells and cell lines. Human tissue-based experiments were used in up to 80% of studies, with various technical approaches and with the most frequently reported being immunohistochemistry (IHC) in both malignancies.

Beyond methodological heterogeneity, the limited molecular and clinicopathological characterization of the investigated populations represents an important finding of this review. Among CRC studies, MMR/MSI status and CMS assignment were reported in only 18.9% and 8.3%, respectively, whereas EBV status and Laurén type were reported in 17.8% and 11.1% of GC studies. Given the distinct immune features associated with CRC CMS and GC molecular and histological classifications, such limited reporting restricts the assessment of subtype-specific tumor–immune interactions. Consequently, the extent to which the described mechanisms are conserved across distinct tumor subtypes remains uncertain. These findings highlight a gap in the primary literature rather than a limitation inherent to the present review.

The substantial methodological heterogeneity of the included studies, together with their limited molecular and clinicopathological stratification, represents a major challenge in interpreting and comparing the available evidence.

The included studies are based on considerably different experimental approaches, with a predominance of in vitro ones. Moreover, the studies included cannot be directly compared, representing a possible source of inconsistency in the reported findings. Another important aspect is the limited representativeness of the cell lines used, with HCT and SWs cell lines being predominantly used for CRC and AGS, MKNs and HGC27 for GC. An important advantage of this methodological approach is the homogeneity and better direct comparison of results but, on the other hand, this reduces the ability to cover the biological diversity of the patients.

CRC experimental models differ in their molecular characteristics and immunogenicity. MC38 is generally considered a relatively immunogenic, checkpoint-responsive murine model, whereas CT26 has a distinct immune phenotype [304]. Human CRC cell lines, including LoVo, RKO, DLD-1, and HCT116, differ in their molecular backgrounds and mismatch-repair status [305]. In GC, AGS and MKN45/MKN28 have been associated with particular molecular subtype features, but such assignments do not establish subtype-specific immune behavior [306]. Therefore, tumor–immune interactions must be interpreted in the context of the selected cell line, experimental model, and host immune competence. Conventional and widely used validation techniques on human samples (IHC, RT-qPCR, WB, IF) suggest that most studies focused on validating candidate biomarkers rather than conducting comprehensive or systems-level experiments. Furthermore, molecular analyses based on public databases were frequently employed, with the results being dependent on certain variables such as the quality of the original datasets or cohort composition, which represents a risk of bias.

Our analysis revealed a predominance of subcutaneous over orthotopic models in both CRC and GC (Table 4). Subcutaneous implantation does not fully reproduce the immune microenvironment of the colonic or gastric mucosa. Because the efficacy of immune checkpoint inhibition depends partly on the spatial distribution of immune cells within tumors, therapeutic responses observed in subcutaneous models may not reliably predict responses in orthotopic tumors or clinical settings [304]. Although these models remain valuable for mechanistic research, complementary validation in orthotopic models and relevant metastatic niches is needed to strengthen translational relevance. Findings involving extracellular vesicles and peritoneal dissemination should likewise be interpreted in the context of the tumor models and anatomical sites investigated, rather than generalized to all GC subtypes.

These methodological approaches cannot fully recapitulate the highly complex in vivo tumor microenvironment, directly influenced by various extratumoral factors (e.g., hormones, cytokines), the absence of a human systemic immune response, and low cellular heterogeneity as a result of cell cultures composed of a single cell type. Mouse experiments overcome some of these limitations but cannot fully replace the human microenvironment.

The limitations of the review process are highlighted by the necessity of excluding some of the research papers. The available pool of studies is extensive for such a complex subject, and we included studies focused mostly on molecules/mechanisms of interest. Because several clinical trials enrolled biomarker-selected populations, their findings may not be generalizable to unselected CRC or GC populations.

Despite the above, the results obtained and published in recent years represent a solid foundation for further studies and in-depth analysis of crosstalk mechanisms between tumor cells and immune cells for their subsequent exploitation. The first steps toward this aim have already been taken in early-phase clinical trials presented in Section 6, and these could represent the future of targeted, highly efficient immunotherapy strategies.

There are numerous gaps in the knowledge of molecular interactions between tumor cells and immune cells within the microenvironment of digestive malignancies. Some of the recently identified molecules involved in tumor progression but with unknown roles are already specified in the last paragraph of Section 5.

The literature describes numerous molecules as important contributors to tumor progression, but the significant impact comes from their coordinated or combined action, not from individual effects. A subset of them has an established impact on tumor microenvironment shaping, but the precise molecular mechanisms underlying their interactions with other cells are unknown. Moreover, a subset of these molecules or mechanisms is extensively studied in one cancer but with little to no evidence for the other one. Although the results are expected to be similar, they may be unpredictable.

The tumor cell–macrophage interactions described in this review revealed some gaps in knowledge. In gastric cancer, further research needs to elucidate the role of the RNF32/SPP1 (osteopontin) axis, including its regulators in both cancers and the intracellular signaling cascades influenced by osteopontin, which remain remarkably less characterized than in colorectal cancer. Given that ADAR1 has been reported to regulate osteopontin and the PKR/EIF2α signaling pathway, it is also important to determine whether these mechanisms are interconnected. Moreover, the impact of ANKRD22 on gastric cancer progression remains to be established, as the current evidence is largely focused on CRC. Further investigations need to focus on the MMP1/PAR1 interaction in GC and on a more comprehensive characterization of its role in CRC. Moreover, the mechanism based on EV-mediated TREM2 transfer between tumor cells and macrophages should be investigated in digestive cancers, as similar results were reported in lung adenocarcinoma. Finally, the regulatory network involving TEAD4, TRIB3 and VEGFA requires further clarification. Since TEAD4 increases TRIB3 levels and increased TRIB3 levels are associated with high VEGFA levels, it remains to be determined whether TEAD4 may directly regulate VEGFA levels.

The tumor cell-CD8+ T-cell interactions described in this review also highlight some knowledge gaps. In gastric cancer, there are knowledge gaps regarding urocortin’s role and its downstream effector pathways, for example, UCN/CCL23, described in colorectal cancer. Furthermore, the relevance of the CCL23/GSK3β axis to tumor progression in digestive cancers should be evaluated. The FSTL3-HIF-1α pathway and the capacity of YWHAH to promote tumor progression via the MAPK/ERK pathway represent future research topics. LPIN3 shows possible roles in digestive cancers, but there are gaps in the literature regarding its modulators and downstream pathways. The transfer of TSHR by EVs to CD8+ T lymphocytes in CRC should also be investigated in GC. Receptor-ligand interactions such as KLRB1-CLEC2B or CD155-TIGIT need to be described in GC and compared with the results already published for CRC. Moreover, the latter can use the PI3K/AKT/mTOR axis to induce T-cell exhaustion, as noted in non-digestive malignancies. In addition, the impact of HECW1 on tumor progression in CRC should be defined.

Finally, various molecules have a similar influence on the tumor microenvironment (for example, M2-like macrophage enrichment, T-cell exhaustion), suggesting a shared intracellular pathway or gene activator that “switches on” all these molecules that drive tumor progression. These shared roots and precise interactions need to be further studied intensively because the tumor microenvironment remains the most important factor that promotes and drives tumor progression and possesses the highest potential for therapeutic success in malignant digestive tumors.

The central evidence emerging from the current literature is that tumor cell–immune cell interactions in colorectal and gastric cancers are governed by a dynamic and highly interconnected ecosystem, rather than by the isolated effects of individual immune cell populations. Tumor cells actively shape the tumor microenvironment by promoting CD8+ T-cell dysfunction while favoring the macrophage M2 phenotype. Moreover, immune cells are highly plastic compartments capable of dynamically adapting to the tumor microenvironment, engaging feedback mechanisms that may progressively favor immune evasion and tumor progression. These observations support a therapeutic strategy of simultaneous modulation of TAMs and CD8+ T-cells rather than targeting either compartment alone, as reflected in most current therapeutic strategies.

## Figures and Tables

**Figure 1:**
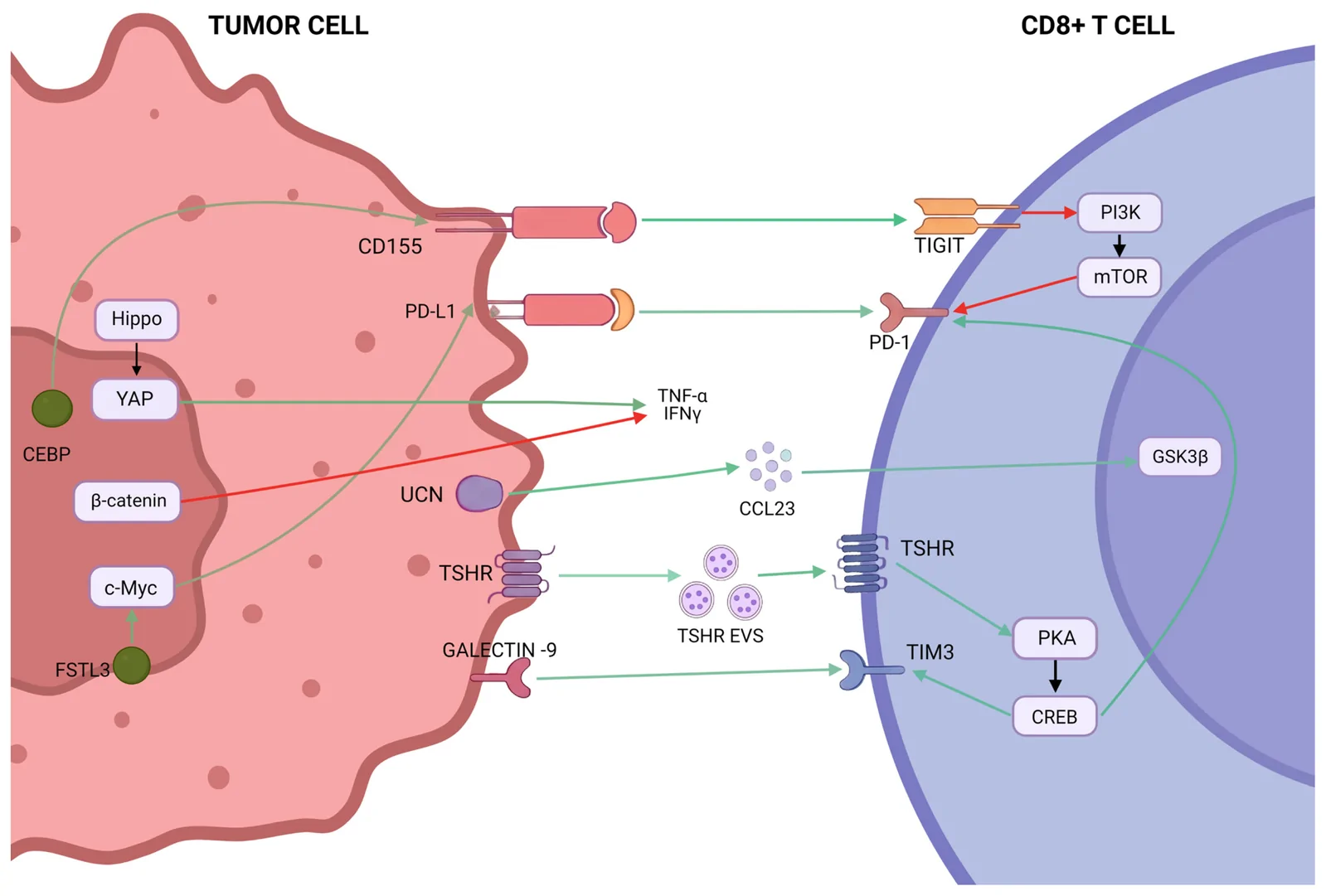
Main molecular interactions between tumor cells (**left**) and CD8+ T lymphocytes (**right**) in colorectal and gastric cancer. The figure focuses on the principal mechanisms mediating direct interactions between cells of interest. Green arrows indicate stimulatory effects; red arrows indicate inhibitory effects; black arrows connect successive steps of signaling pathways or indicate the transfer route of molecules. The illustrated mechanisms are not assigned to a particular molecular subtype. Additional components are presented in Supplementary Figure S1. Created in BioRender. Andrei, P. (2026) https://BioRender.com/8yqghug.

**Figure 2:**
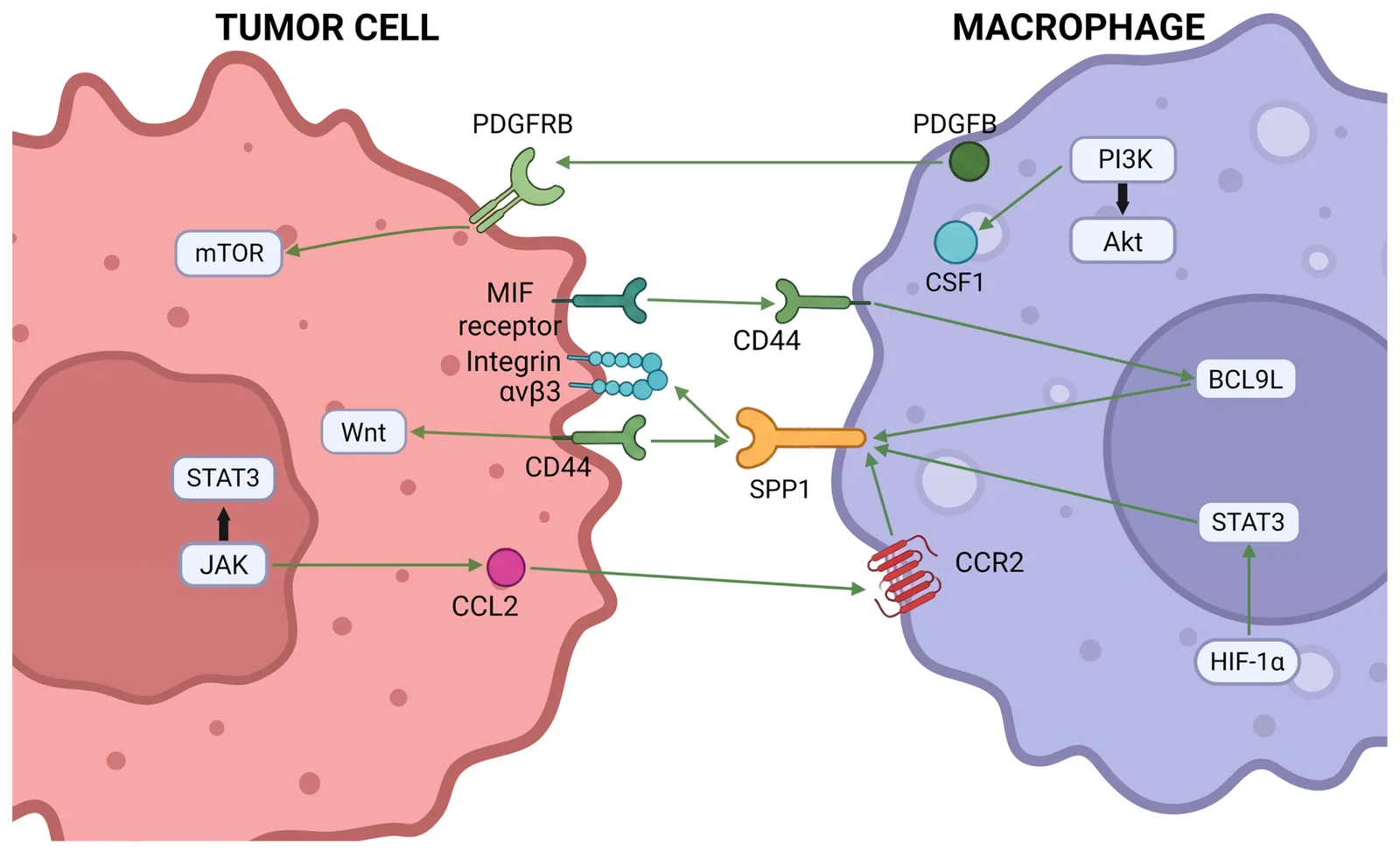
Main molecular interactions between tumor cells (**left**) and SPP1+ macrophages (**right**) in colorectal and gastric cancer. The figure focuses on the principal mechanisms mediating direct interactions between these cells, with particular emphasis on osteopontin-associated signaling. Green arrows indicate stimulatory effects; black arrows connect successive steps of signaling pathways. Molecular subtypes were not consistently specified in the underlying studies; therefore, the illustrated mechanisms are not assigned to a particular molecular subtype. Additional components are presented in Supplementary Figure S2. Created in BioRender. Andrei, P. (2026) https://BioRender.com/2cit6np.

**Figure 3:**
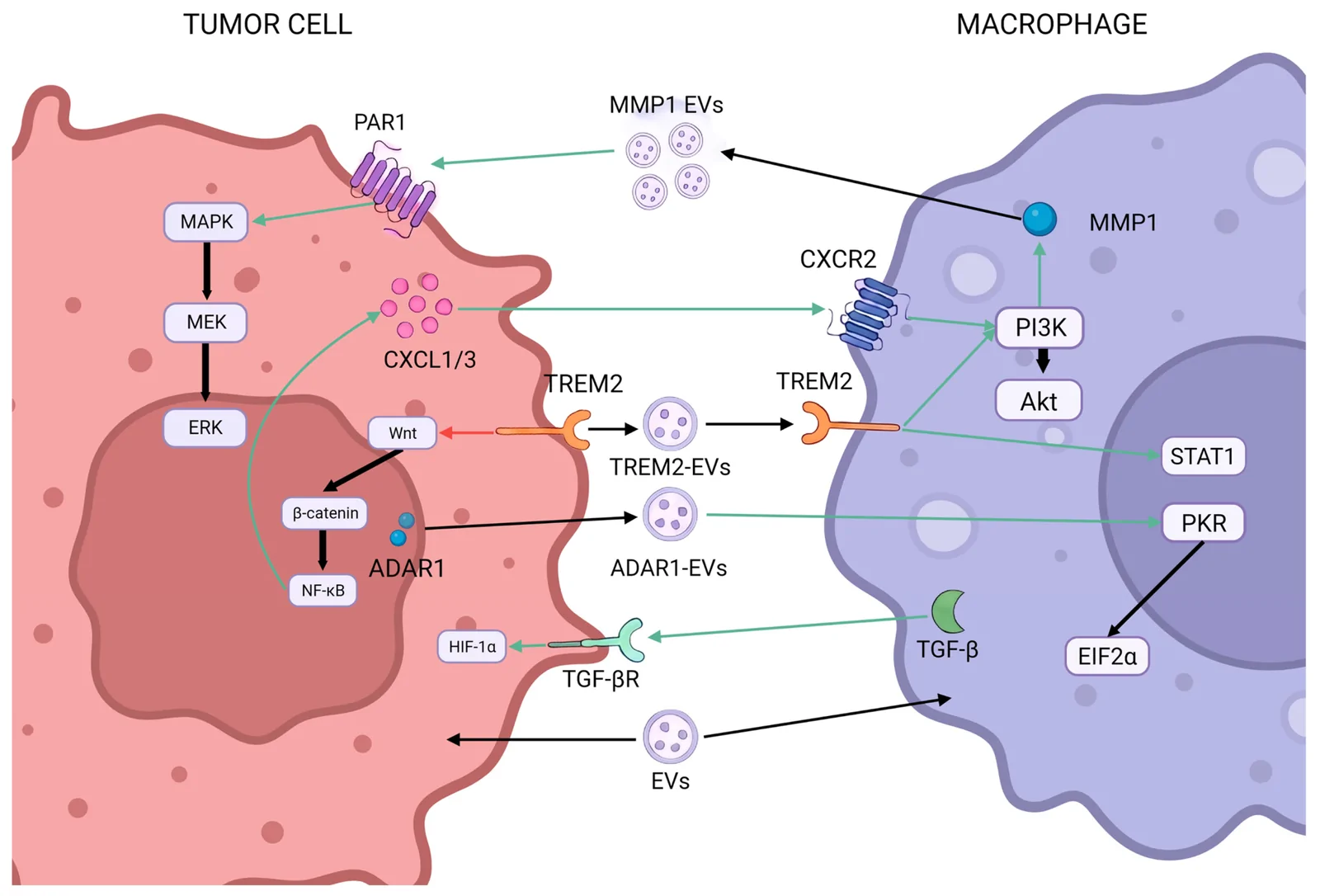
Main molecular interactions between tumor cell (**left**) and macrophages (**right**) in colorectal and gastric cancer. The figure focuses on the mechanisms mediating direct interactions between these cells, including EVs-mediated communication. Green arrows indicate stimulatory effects; red arrows indicate inhibitory effects; black arrows connect successive steps of signaling pathways or indicate the transfer route of molecules. The illustrated mechanisms are not assigned to a particular molecular subtype. Additional intracellular modulators are presented in Supplementary Figure S3. Created in BioRender. Andrei, P. (2026) https://BioRender.com/aavlvgp.

**Table 1:** Detailed interactions through EVs between tumor cells and macrophages in GC and CRC; M1P/M2P—M1-like /M2-like polarization; EMT—epithelial-mesenchymal transition; ANG—angiogenesis; TG—tumor growth; CTR—chemotherapy resistance; PM—metastasis promotion; NGFR—nerve growth factor receptor.

Tumor	Name	Origin	Target	Pathway	Effect	Reference
CRC	miR-25-3p miR-130b-3p miR-425-5p	TC	M	PTEN/PI3K/AKT	M2P EMT ANG	[228]
CRC	circVCP	TC	M	miR-9-5p/NRP1	M2P, TP	[229]
*p* > dMMR CRC	miR-203a-3p	TC	M	PTEN/AKT	M2P TP	[230]
CRC	circ-0034880	TC	M	-	PM	[225]
CRC	HSP90B1	TC	M	-	M2P	[227]
CRC	lnc-HOXB8-1:2	TC	M	lnc-HOXB8-1:2/hsa-miR-6825-5p/CXCR3 axis	M2P	[231]
CRC	miR-188-5p	TC	M	TIMP2/3	PM, M2P	[232]
CRC	miR-372-5p			PTEN/AKT/NF-kB	M2P, EMT, PM (via CXCL12), CD8+ cells decrease, PD-L1 increase	[233,234]
CRC	microRNA-106b-5p	EMT-TC	M	PDCD4/PI3Kg/AKT/mTOR axis	M2P	[235]
CRC	circPOLQ	TC	M	miR-379–3p/IL-10/ STAT3 axis	M2P	[236]
CRC	ITGαvβ5	TC	M	P38/STAT1/CXCL12	PM	[237]
CRC	LINC01615	TC	M	RBMX-EZH2	M2P	[226]
CRC	Ferritin heavy chain 1	M	TC	-	TP	[238]
CRC	THBS2	TC	M	Wnt/β-catenin	M2P	[239]
CRC	MiR-769-5p	M	TC	MAPK1	CTR	[240]
CRC	M2-like-EVs	M2-like	TC	FAM83A	EMT	[241]
CRC	CASC19	M1-like	TC	miR-410-3p	Inhibition of proliferation and migration	[242]
GC	miR-92a-3p	TC	M	ERK	M2P, PD-L1 increase	[243]
GC	miR-541-5p	TC	M	DUSP3/JAK2/STAT3	M2P, TP, invasion	[244]
GC	circATP8A1/miR-1-3p	TC	M	STAT6	TP, migration, M2P	[245]
GC	miR-519a-3p	TC	M	DUSP2-MAPK/ERK axis	PM, M2P, ANG	[246]
Diffuse GC	let-7g-5p	TC	M	JAK2/STAT3	M2P	[247]
GC	HMGB1	TC	M	NF-kB	M2P	[248]
GC	NGFR	TC	M	NGFR/RIPK1	M2P	[249]
GC	miR-151a-5p	TC	M	DUSP8/MAPK/ELK1	Increase LAG3 expression	[250]
GC	MALAT1	M	TC	β-Catenin/HIF-1α upregulation	TP	[251]
GC	ELFN1-AS1	TC	M	miR-4644/PKM axis	M2P, TP, PM	[252]
GC	miR-487a	M	TC	TIA1	TP	[253]

**Table 2:** Main molecular mechanisms mediating interactions between TCs, TAMs and CD8+ T-cells in CRC and GC. Mechanistic effects were not systematically compared across molecular subtypes; therefore, their transferability between subtypes remains unestablished.

Mechanism	Model and Source	Subtype Reported	Reported Mechanistic Effect	References
CD155-TIGIT	CRC/GC tissue/TCGA/CT26 mice	CRC [57]: MSS/MSI; CMS1–4. GC: not reported	CD155–TIGIT suppresses CD8+ PI3K/AKT/NF-κB (CRC) and AKT/mTOR (GC)	[56,57,61,62,64]
CLEC2B-KLRB1	CRC tissue, MC38-OVA mouse model	MSS cohort	Decreases CD8+ PI3K/AKT and ERK activity—exhaustion	[65]
UCN-CCL23	CRC TCGA, tissue, HCT116/HT29	CRC: left/right location only; no molecular subtype	UCN upregulates CCL23—CD8+ T-cell exhaustion	[101]
TSH-TSHR	CRC tissue, MC38/CMT93 mice	MSS/MSI reported	TSHR-PKA/CREB—exhaustion	[73]
Gal-9-TIM-3	CRC/GC tissues, mouse model, TCGA/GEO	[78] Mostly pMMR; [80] MSI-H; [81] CMS1/CMS4 enrichment	Gal-9/TIM-3 promotes CD8+ dysfunction	[76,77,78,79,80,81,82]
PD-1-PD-L1	CRC tissue/TCGA, MC38 mice [39]; CMS4-like organoids/mice [87]	[39] FSTL3-high enriched in MSI-H; [87] CMS4-like, KRAS G12D	PD-L1 suppresses CD8+ immunity	[39,74,87]
CCL2-CCR2	CRC tissue, TCGA/GEO, tumor–TAM co-culture, mouse models [137,213,214,215,216,217,218,219,220,221,222]	Not reported	CCL2-CCR2 recruits macrophages and promote a M2-like state	[137,211,212,213,214,215,216,217,218,219,221,222]
MMP1-PAR1	HT-29, Caco-2 cells TAM–TC co-culture Mouse xenograft	Not reported	MMP1–PAR1/ERK favors TC proliferation	[188]
TREM2	CRC/GC tissues, HGC-27/MKN45/HT29/MC38, mouse models	[158] GC EBV/MSI/GS/CIN; TREM2-high enriched in EBV	TREM2 upregulates PD-L1/CCL8 level—immunosuppression	[152,154,157,158]
SPP1	CRC/GC tissue, mice	[131] Diffuse GC (Laurén); [132,143] MSI/MSS; [145] KRAS	SPP1+ TAMs drive CD44/Wnt, JAK/STAT3, invasion and CD8+ suppression	[131,132,135,141,143,145]
TGF-β- TGF-βR	CRC HCT116/CT26–TAM assays; NOD-SCID/BALB/c mice	Not reported	TGF-β upregulates TC HIF-1α/TRIB3/Wnt	[164]
TRIB3	CRC/GC tissues/TCGA, HCT-116/CT26/SW480, co-culture, mice	Not reported	TRIB3 stimulates Wnt/VEGFA, angiogenesis and ferroptosis resistance	[113,164,165]
GPX4-SLC7A11	CRC TCGA/GEO, human tissues, HCT116/SW480/HT-29/LoVo, xenografts	Not reported	GPX4/SLC7A11 induces ferroptosis resistance	[113,169]

**Table 3:** Clinical trials focused on tumor cell–immune cell interaction mechanisms and their main characteristics; RNF—results not found; A—active; R—recruiting; NYR—not yet recruiting; T—terminated; NBSS—no biomarker-selected subgroup; WT—wild type; for other abbreviations, see the “Abbreviations” section.

Clinical Trial/Phase/Status	Agents (Mechanism of Action)	Inclusion Criteria	Biomarker-Based Selection/Reported Biomarker Status	Primary Aims	Results	Reference
NCT04354246 I-C	COM902 (TIGIT inhib.) + COM701 + Pembrolizumab	Advanced CRC	MSS	ST, PK	SD = 35% DCR = 40%	[276]
NCT06784947 II-A	Tiragolumab (TIGIT inhib.)	Metastatic colonic adenocarcinoma	MSS	ORR	RNF	-
NCT05009069 II-A	Atezolizumab + Tiragolumab (TIGIT inhib.)	Locally advanced adenocarcinoma of rectum	MSS * PD-L1 expression = 2%	CR	CR = 35.7% 1-year	[277]
NCT04929223 I-R	ATB: Atezolizumab + Tiragolumab (TIGIT inhib.) + Bevacizumab AT: Atezolizumab + Tiragolumab	Metastatic CRC	NBSS	ORR	RNF	-
PURPLE trial: EU-CT Number: 2022-503044-40 II-A	Atezolizumab + Tiragolumab (TIGIT inhib.)	Liver metastases of CRC	MSS	CR	RNF	[278]
NCT05329766 II-A	Domvanalimab (TIGIT inhib.) + Zimberelimab + FOLFOX	Advanced or metastatic Her2-negative gastric cancer	* MSI status: High (2%), Low (10%), Stable (76%), Unknown (12%) * HER2-negative * PD-L1 (TAP score) negative: 27.5% Positive: 72.5%	ORR AE	ORR = 59% CR = 7% PR = 51% DCR = 93%	[279]
NCT04666688 I-C	LYT200 (Galectin-9 inhib.)	Advanced or metastatic solid tumors, including CRC	NBSS	ST, PK, PD	No DLTs or SAEs 1 case of SD	[280]
NCT04691375 Ib-C	PY314 (Anti-TREM2) PY314 + pembrolizumab	Advanced solid tumors (included colorectal and gastric carcinoma)	MSI CRC	AE DLT PK PD	RNF	-
NCT06877533-TRM-010 Ib-T	EOS006215 (Anti-TREM2) EOS006215 + other anticancer agents	Advanced solid tumors	NBSS	DLT AE SAEPD	RNF	-
NCT01390818 I-C	Voxtalisib-SAR245409 (PI3K and mTOR inhib.)	Advanced solid tumors—including CRCs	KRAS mutation present	DLT	RR = 0%	[281]
NCT04495621 II-C	MEN1611 (PI3K inhib.) + Cetuximab	Metastatic CRC	N-K-RAS WT BRAF WT	DLT, ORR	Total = 14 SD = 57.1% PD = 28.6% PR = 7.1% CR = 7.1%	[282]
NCT01364844 I-C	DS-7423 (PI3K/mTOR Inhib.)	Advanced Solid Tumors—CRC included	NBSS	AE	RNF	-
NCT01252628 II-C	PX-866 (PI3K inhib.)	Metastatic CRC + Head and Neck Squamous cell carcinoma	Excluded: KRAS mutation in codon 12 or 13	Antitumor effect	Total = 42 CR = 0% PR = 2.4% (1) DCR = 40.5% PFS = 59 days	[283]
NCT01719380 Ib/II-C	BYL719 (PI3K inhib.) + LGX818 (BRAF inhib.) + Cetuximab	Metastatic CRC	BRAF V600-mutant CRCs included	PFS	TOTAL = 28 PFS-4.1 to 5.4 months (based on dose-phase Ib) ORR-26.9% (phase II)	[284]
NCT01576666 I-C	Buparlisib (BKM120-PI3K inhib.) + Sonidegib (LDE225-Hedgehog inhib.)	Advanced tumors-metastatic CRCs and GCs included	NBSS	DLT	No results, patients discontinued	-
NCT02465060 II-A	GSK2636771 (PI3K beta inhib.)	Solid tumors—Digestive malignancies included	dMMR (subprotocol Z1D) BRAF-mutant (subprotocol H) HER2 amplification (subprotocol J)	ORR	No results available for the subprotocols of interest	[285]
NCT01613950 II-C	BYL719 (PI3K inhib.) + AUY922 (HSP90 inhib.)	Advanced or metastatic GC	HER2 overexpression	DLT	RNF	-
NCT01260701 II-C	MK2206 (AKT pathway inhib.)	Advanced gastric cancer	NBSS	OS	OS = 5 months CR + PR = 1.5%	[286]
NCT02240212 I-C	Afuresertib (AKT pathway inhib.)	Metastatic or locally advanced gastric cancer	HER2 negative	SAE	RNF	-
NCT01896531 II-C	Ipatasertib (AKT pathway inhib.) + mFOLFOX6	Advanced or metastatic gastric cancer	HER2 negative	PFS	PFS = 6.57 months OS = 11.96 months ORR = 52.1%	[287]
NCT01802320 II-C	MK2206 (AKT pathway inhib.)	Metastatic or advanced CRC	BRAF and KRAS WT	ORR	ORR = 0% OS = 6.8 months PFS = 1.87 months	[288]
NCT06301386 II-NYR	Everolimus (mTOR pathway inhib.) + PD-1	Advanced CRC	NBSS	ORR	RNF	-
NCT01154335 I-C	Everolimus (mTOR pathway inhib.) + OSI-906	Metastatic CRC	KRAS mutated or WT	MTD	PFS = 1.6–3.6 months (based on dose) OS = 3.3–5.1 weeks (based on dose)	[289]
NCT01047293 I/II-C	Everolimus (mTOR pathway inhib.) +Bevacizumab + FOLFOX	Advanced and metastatic CRC	NBSS	PFS	PFS at 6 months = 87%	[290]
NCT03082833 II-T	AZD2014 (mTOR pathway inhib.)	Gastric adenocarcinoma	NBSS	ORR	Lack of efficacy	-
NCT00985192 II-C	Everolimus (mTOR pathway inhib.)	Gastric + esophageal adenocarcinoma	NBSS	DCR	DCR = 40% OS = 3.4 months PFS = 1.8 months	[291]
NCT04616183 I/II-A	A: LY3214996 (ERK 1/2 inhib.) + cetuximab B: LY3214996 + cetuximab + abemaciclib	Advanced and metastatic CRC	KRAS, NRAS, BRAF WT * pMMR/MSS	CR + PR	A/B = ORR = 5.3%/15.0% PFS = 1.8/3.6 months OS = 7/14 months	[292]
NCT02857270 I-C	LY3214996 (Anti-ERK1/2) −/+ other anticancer agents	Metastatic CRC and other malignancies	BRAF V600E	DLT	RNF	[293]
NCT02277093 II-T	Pacritinib (JAK/STAT inhib.)	Refractory CRC	* 8/11 (73%) enrolled patients present RAS mutations	PFS	PFS = 1.91 months CR, PR = 0% Increased side effects	[294]
NCT02119676 II-T	Ruxolitinib (JAK inhib.) + regorafenib (kinase inhib.)	Metastatic CRC	* KRAS mutated (no/yes/unknown) 41.4%/55.2%/1.1% * BRAF mutated (no/yes/unknown) 44.7%/2.3%/51.7%	OS	OS = 11.4 months PFS = 3.5 months ORR = 2.7%	[295]

* reported as baseline or exploratory characteristic, rather than used as eligibility criteria.

**Table 4:** Main methodological differences between gastric and colorectal cancer TME studies, expressed as the total number and proportion of studies reporting each methodology. Categories are not mutually exclusive, as individual studies may employ more than one implantation model or metastatic site. Percentages were calculated using the total number of studies for each cancer type (CRC, n = 132; GC, n = 45), except for tumor implantation sites and metastatic niches, for which the denominators were the numbers of studies using mouse models (CRC, n = 80; GC, n = 25).

Methodology	CRC n = 132		GC n = 45
Online biodatabases	62 (46.9%)		23 (51.1%)
Cell lines	97 (73.4%)		33 (73.3%)
-SWs	52 (39.4%)	AGS	18 (40%)
-HCTs	59 (44.7%)	SGC7901	7 (15.5%)
-LS174T	2 (1.5%)	MKNs	19 (42.2%)
-Lovo	25 (18.9%)	HGC27	15 (33.3%)
-RKO	13 (9.8%)	N87	1 (2.2%)
-DLD1	12 (9.1%)	SNU	3 (6.6%)
-Colon-26	1 (0.7%)	BGC823	7 (15.5%)
CT-26	18 (13.6%)	MGC803	6 (13.3%)
CMT93	3 (2.2%)	SNU-1	1 (2.2%)
NCM460	2 (1.5%)	OCUM-1	1 (2.2%)
MC-38	11 (8.3%)		
HTs	23 (17.4%)		
CaCo-2	9 (6.8%)		
Molecular analysis	88 (66.6%)		31 (68.9%)
Genetic engineering	66 (50%)		27 (60%)
Cellular functional assays	62 (46.9%)		20 (44.4%)
Metabolism	10 (7.5%)		4 (8.9%)
Co-culture	11 (8.3%)		4 (8.9%)
Xenograft experiments-mouse	80 (60.6%)		25 (55.5%)
Tumor location—subcutaneous	61 (76.2%)		12 (48%)
Tumor location—orthotopic	10 (12.5%)		2 (8%)
Metastatic niche—liver	17 (21.2%)		1 (4%)
Metastatic niche—peritoneum	4 (5%)		4 (16%)
Human tissue-based experiments	96 (72.7%)		36 (80%)
IHC	67 (50.7%)		25 (55.5%)
WB	17 (12.9%)		5 (11.1%)
IF	27 (20.4%)		6 (13.3%)
FC	5 (3.8%)		5 (11.1%)
RT-qPCR	28 (21.2%)		6 (13.3%)
ELISA	5 (3.8%)		2 (4.4%)
IB	1 (0.7%)		1 (2.2%)
RNA-seq	18 (13.6%)		5 (11.1%)
MMR/MSI status reported	25 (18.9%)	EBV status	8 (17.8%)
CMS assignment	11 (8.3%)	Laurén type	5 (11.1%)
Tumor laterality with BRAF/RAS	0 (0%)	HER2/CPS reported	3 (6.7%)

## Data Availability

No new data were created or analyzed in this study. Data sharing is not applicable to this article.

## Supplementary Materials

The following supporting information can be downloaded at: https://www.mdpi.com/article/10.3390/ijms27209346/s1.

## Abbreviations

The following abbreviations are used in this manuscript:

**Abbreviation**	**Definition**
A	Active
Act1	NF-kappa B activator 1
ADAP1	ArfGAP with dual PH domains 1
AE	Adverse event
ANG	Angiogenesis
ANKRD22	Ankyrin repeat domain-containing protein 22
CAMSAP2	Calmodulin-regulated spectrin-associated protein 2
CR	Complete response
CRC	Colorectal cancer
CTR	Chemotherapy resistance
CXCR/L	CXC motif chemokine receptor/ligand
DCR	Disease control rate (SD + CR + PR)
DLT	Dose-limiting toxicity
ELISA	Enzyme-linked immunosorbent assay
EMT	Epithelial-mesenchymal transition
EVs	Extracellular vesicles
FC	Flow cytometry
FOXC1	Forkhead box protein C1
FSTL3	Follistatin-like 3
GC	Gastric cancer
GPX4	Glutathione peroxidase 4
HIF-1α	Hypoxia-inducible factor 1-alpha
IB	Immunoblotting
IF	Immunofluorescence
IFI35	Interferon-induced protein 35
IFN-α/γ	Interferon alpha/gamma
IHC	Immunohistochemistry
IL	Interleukin
LPIN3	Lipin 3
M1P/M2P	M1-like/M2-like polarization
MCAM	Melanoma cell adhesion molecule
MCL-1	Myeloid cell leukemia 1
MDSCs	Myeloid-derived suppressor cells
MFSD2A	Major facilitator superfamily domain-containing 2
MIF	Macrophage migration inhibitory factor
MMPs	Matrix metalloproteinases
MTD	Maximum tolerated dose
NETs	Neutrophil extracellular traps
NGFR	Nerve growth factor receptor
NYR	Not yet recruiting
ORR	Overall response rate
OS	Overall survival
PD	Progressive disease
PFS	Progression-free survival
PK	Pharmacokinetics
PM	Promotes metastasis
PR	Partial response
R	Recruiting
RNA-seq	RNA sequencing
RNF32	RING finger protein 32
RNF	Results not found
ROS	Reactive oxygen species
RR	Response rate
RT-qPCR	Reverse transcription quantitative PCR
SAE	Serious adverse event
SD	Stable disease
ST	Safety and tolerability
T	Terminated
Tpex	Precursor exhausted CD8+ T-cell
Tex	Exhausted CD8+ T-cells
TAFs	Tumor-associated fibroblasts
TAMs	Tumor-associated macrophages
TCs	Tumor cells
TG	Tumor growth
TIMP2	Tissue inhibitor of metalloproteinase-2
TME	Tumor microenvironment
TNF-α	Tumor necrosis factor alpha
TP	Tumor progression
Tregs	Regulatory T-cells
TREM2	Triggering receptor expressed on myeloid cells 2
TRIB3	Tribbles pseudokinase 3
TRIM26	Tripartite motif-containing protein 26
TSHR	Thyroid-stimulating hormone receptor
UCN	Urocortin
USP48	Ubiquitin-specific peptidase 48
VEGF	Vascular endothelial growth factor
WB	Western blot
YTHDF1	YTH N6-methyladenosine RNA-binding protein 1
ZNF3	Zinc finger protein 3
