# Supplementary material for: Tumor-Centric Immune Crosstalk in Colorectal and Gastric Cancer: Molecular Mechanisms Linking Tumor Cells to Macrophages and CD8+ T Lymphocytes
Source: Int J Mol Sci. 2026 Oct 21;27(20):9346. doi: 10.3390/ijms27209346 (PMC13704946; doi:10.3390/ijms27209346)
Supplement: Supplementary file 1 [file ijms-27-09346-s001.zip › ijms-4608565-supplementary.pdf]

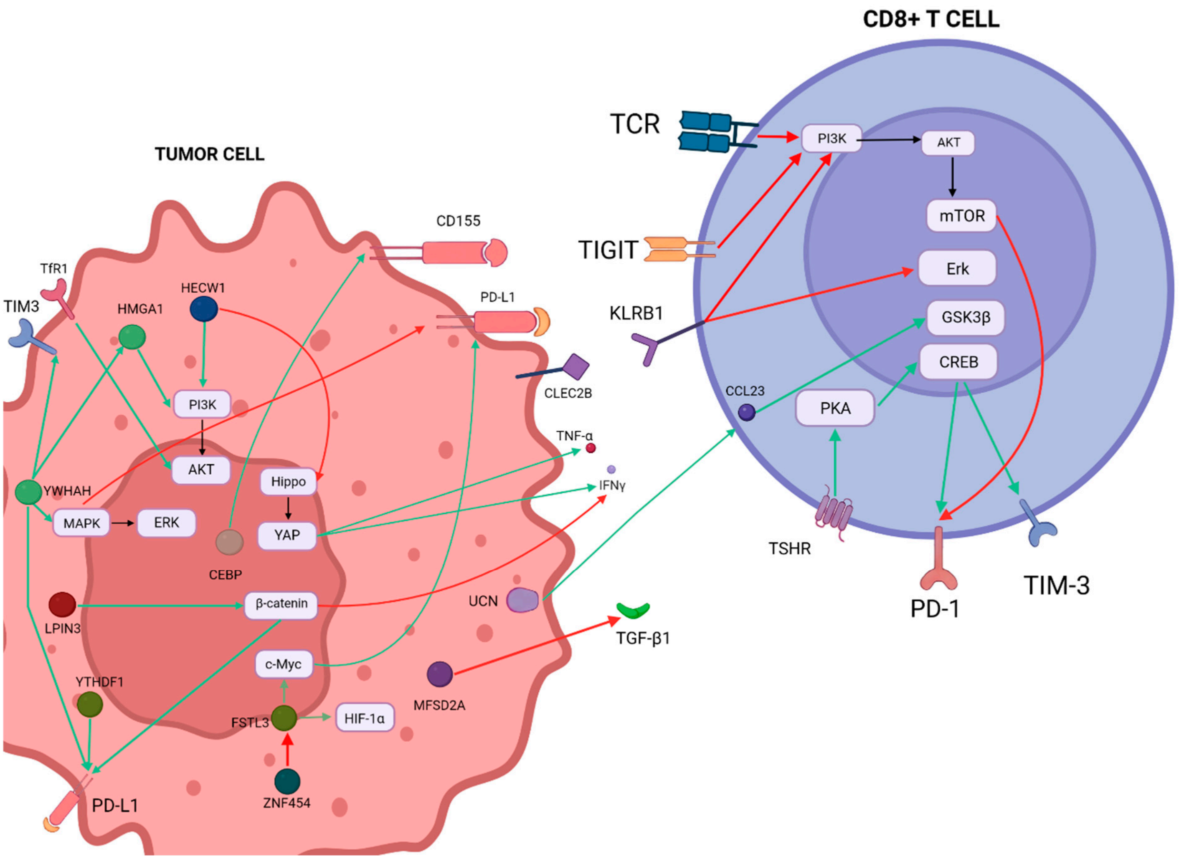

Figure S1. Additional molecular regulators and signaling pathways involved in tumor cell (left) and CD8+ T-lymphocyte (right) crosstalk in colorectal and gastric cancer. The figure expands on Figure 1 by illustrating intracellular pathways and regulatory molecules associated with intercellular interaction. Green arrows indicate stimulatory effects; red arrows indicate inhibitory effects; black arrows connect successive steps of signaling pathways. Molecular subtypes were not consistently specified in the underlying studies; therefore, the illustrated mechanisms are not assigned to a particular molecular subtype and should not be interpreted as universally applicable across subtypes. *Created in BioRender. Andrei, P. (2026) https://BioRender.com/9vn1hr7*

Abbreviations: AKT, protein kinase B; CCL23, C–C motif chemokine ligand 23; CD155, cluster of differentiation 155; CEBP, CCAAT/enhancer-binding protein; CLEC2B, C-type lectin domain family 2 member B; CREB, cAMP response element-binding protein; ERK, extracellular signal-regulated kinase; FSTL3, follistatin-like 3; GSK3β, glycogen synthase kinase 3β; HECW1, HECT, C2 and WW domain-containing E3 ubiquitin protein ligase 1; HIF-1α, hypoxia-inducible factor 1-alpha; HMGA1, high mobility group AT-hook 1; IFN-γ, interferon-gamma; KLRB1, killer cell lectin-like receptor B1; LPIN3, lipin 3; MAPK, mitogen-activated protein kinase; MFSD2A, major facilitator superfamily domain-containing protein 2A; mTOR, mechanistic target of rapamycin; PD-1, programmed cell death protein 1; PD-L1, programmed death-ligand 1; PI3K, phosphoinositide 3-

kinase; PKA, protein kinase A; TCR, T-cell receptor; TfR1, transferrin receptor 1; TGF-β1, transforming growth factor beta 1; TIGIT, T-cell immunoreceptor with immunoglobulin and ITIM domains; TIM-3, T-cell immunoglobulin and mucin domain-containing protein 3; TNF-α, tumor necrosis factor-alpha; TSHR, thyroid-stimulating hormone receptor; UCN, urocortin; YAP, Yes-associated protein; YTHDF1, YTH domain-containing family protein 1; YWHAH, tyrosine 3-monooxygenase/tryptophan 5-monooxygenase activation protein eta (14-3-3η); ZNF454, zinc finger protein 454.

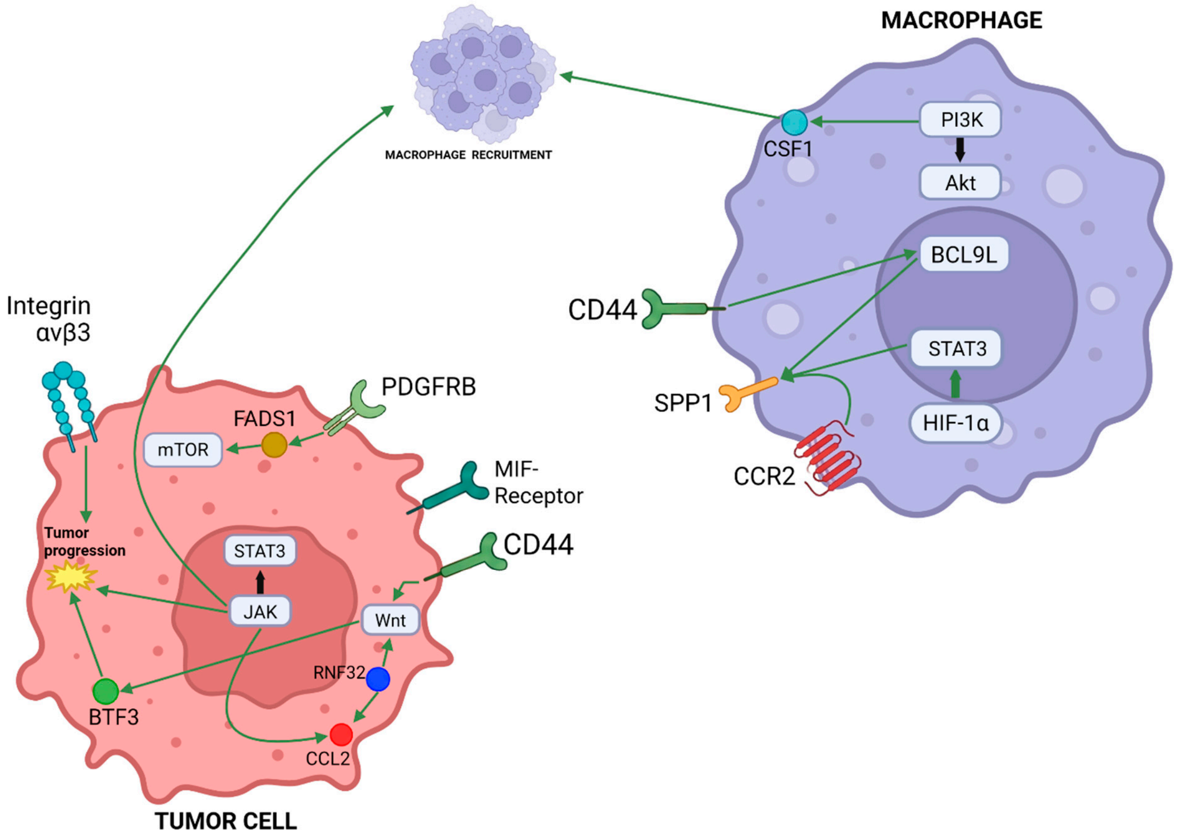

Figure S2. Additional molecular regulators and signaling pathways associated with SPP1-mediated tumor cell (left) and macrophage (right) crosstalk in colorectal and gastric cancer. The figure expands on Figure 2 by illustrating intracellular pathways associated with SPP1 expression, tumor progression, and macrophage recruitment. Green arrows indicate stimulatory effects; black arrows connect successive steps of signaling pathways. The illustrated mechanisms are not assigned to a particular molecular subtype and should not be interpreted as universally applicable across subtypes. *Created in BioRender. Andrei, P. (2026) https://BioRender.com/jt2n9ds*

Abbreviations: AKT, protein kinase B; BCL9L, B-cell CLL/lymphoma 9-like protein; BTF3, basic transcription factor 3; CCL2, C–C motif chemokine ligand 2; CCR2, C–C motif chemokine receptor

2; CD44, cluster of differentiation 44; CSF1, colony-stimulating factor 1; FADS1, fatty acid desaturase 1; HIF-1α, hypoxia-inducible factor 1-alpha; JAK, Janus kinase; MIF, macrophage migration inhibitory factor; mTOR, mechanistic target of rapamycin; PDGFRβ, platelet-derived growth factor receptor beta; PI3K, phosphoinositide 3-kinase; RNF32, RING finger protein 32; SPP1, secreted phosphoprotein 1 (osteopontin); STAT3, signal transducer and activator of transcription 3; Wnt, Wingless-related integration site; αvβ3, integrin alpha-v beta-3.

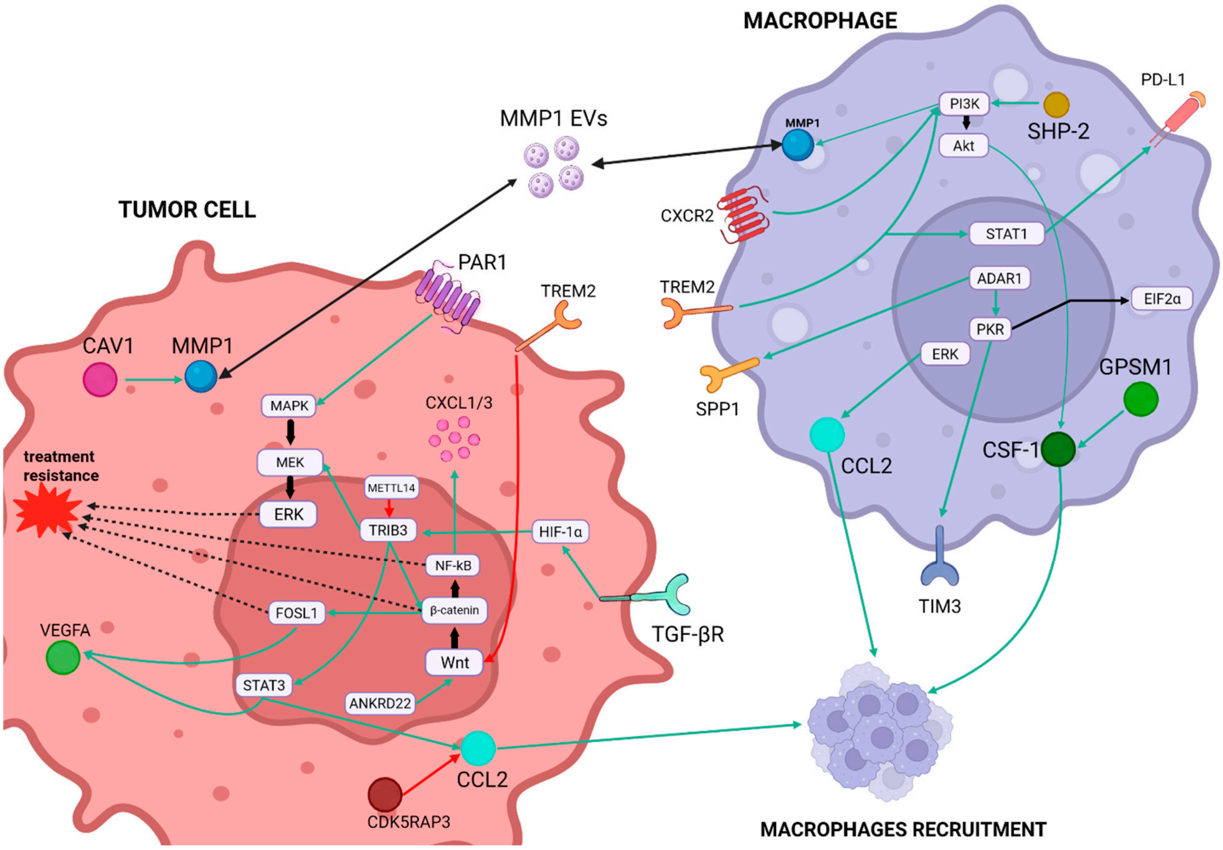

Figure S3. Additional molecular regulators and signaling pathways involved in tumor cell (left) and macrophage (right) crosstalk in colorectal and gastric cancer. The figure expands on Figure 3 by illustrating intracellular pathways associated with intercellular communication, treatment resistance, and macrophage recruitment. Green arrows indicate stimulatory effects; red arrows indicate inhibitory effects; solid black arrows connect successive steps of signaling pathways or indicate molecular transfer routes. Dashed black arrows indicate contributions to treatment resistance, represented by the red starburst. Molecular subtypes were not consistently specified in the underlying studies; therefore, the illustrated mechanisms are not assigned to a particular molecular subtype and should not be interpreted as universally applicable across subtypes. *Created in BioRender. Andrei, P. (2026) https://BioRender.com/4hhq7lw*

Abbreviations: ADAR1, adenosine deaminase acting on RNA 1; AKT, protein kinase B; ANKRD22, ankyrin repeat domain-containing protein 22; CAV1, caveolin 1; CCL2, C–C motif chemokine ligand 2; CDK5RAP3, CDK5 regulatory subunit-associated protein 3; CSF1, colony-stimulating factor 1; CXCL1/3, C–X–C motif chemokine ligands 1 and 3; CXCR2, C–X–C motif chemokine receptor 2; eIF2α, eukaryotic translation initiation factor 2 subunit alpha; ERK, extracellular signal-regulated kinase; EVs, extracellular vesicles; FOSL1, FOS-like antigen 1; GPSM1, G-protein signaling modulator 1; HIF-1α, hypoxia-inducible factor 1-alpha; MAPK, mitogen-activated protein kinase; MEK, mitogen-activated protein kinase kinase; METTL14, methyltransferase-like 14; MMP1, matrix metalloproteinase 1; NF-κB, nuclear factor kappa B; PAR1, protease-activated receptor 1; PD-L1, programmed death-ligand 1; PI3K, phosphoinositide 3-kinase; PKR, protein kinase R; SHP-2, Src homology 2 domain-containing protein tyrosine phosphatase 2; SPP1, secreted phosphoprotein 1 (osteopontin); STAT1/3, signal transducer and activator of transcription 1 and 3; TGF-βR, transforming growth factor beta receptor; TIM3, T-cell immunoglobulin and mucin domain-containing protein 3; TREM2, triggering receptor expressed on myeloid cells 2; TRIB3, tribbles pseudokinase 3; VEGFA, vascular endothelial growth factor A; Wnt, Wingless-related integration site.
